# Advances and Future Directions in Antibody–Drug Conjugates: From Paradigm Shifts to Data-Driven Design

**DOI:** 10.3390/cancers18132102

**Published:** 2026-06-28

**Authors:** Smita Kumari, Lillian M. Cool, Elizabeth Howard, Jogendra Singh Pawar

**Affiliations:** 1 Division of Medicinal Chemistry and Pharmacognosy, College of Pharmacy, The Ohio State University, 500 W. 12th Ave., Columbus, OH 43210, USAcool.38@buckeyemail.osu.edu (L.M.C.); 2 Neuroscience Program, College of Arts and Sciences, The Ohio State University, 230 North Oval Mall, Columbus, OH 43210, USA; 3 The Ohio State University Comprehensive Cancer Center—Arthur G. James Cancer Hospital and Richard J. Solove Research Institute, 460 W. 10th Ave., Columbus, OH 43210, USA

**Keywords:** antibody–drug conjugates (ADCs), linkers, payloads, conjugation technologies, artificial intelligence, data-driven design, bispecific antibodies

## Abstract

**Background**: Antibody–drug conjugates (ADCs) have evolved from early heterogeneous constructs into a mature therapeutic platform with exponential clinical relevance. This review highlights recent advances in ADC design and development, with emphasis on antigen selection, antibody engineering, linker and payload innovation, site-specific conjugation, clinical translation, toxicity, resistance, and emerging data-driven approaches. **Methods**: The review draws on the literature published from 2019 to the recent clinical and regulatory developments relevant to approved and late-stage ADCs, emphasizing the advances in target biology, antibody formats, linker chemistry, payload classes, conjugation technologies, developability assessment, and computational or artificial intelligence-assisted design strategies. **Results**: ADC development has evolved with improved target selection, enhanced internalization and tumor selectivity, and the use of engineered, bispecific, biparatopic, and fragment-based antibody formats. Linker and payload innovation has expanded beyond traditional microtubule inhibitors to include topoisomerase I inhibitors, DNA-damaging agents, and emerging dual-payload or non-cytotoxic strategies. Site-specific conjugation and improved control of drug-to-antibody ratio have increased stability, pharmacokinetic performance, and manufacturability. Clinically, ADCs are being used across a broader range of malignancies and treatment settings, although toxicities and resistance mechanisms remain an important limitations. Computational methods and artificial intelligence are increasingly being explored for target discovery, molecular optimization, toxicity prediction, and model-informed clinical development. **Conclusions**: ADCs are transitioning toward a more integrated, design-driven platform in which antigen biology, antibody format, chemistry, and computational prediction are jointly optimized. Future progress will depend on improved standardization, biomarker-guided development, and interdisciplinary approaches to enhance its therapeutic index and expand its applications beyond oncology.

## Simple Summary

Antibody–drug conjugates are an important and rapidly evolving class of targeted cancer treatments that combine the precision of antibodies with the killing power of anticancer drugs. This review highlights how recent advances in target selection, antibody engineering, linker design, payload choice, and conjugation methods are improving the effectiveness and safety of these therapies. It also discusses current challenges such as toxicity, resistance, and manufacturing complexity, as well as the growing role of computational and artificial intelligence-based methods in guiding future development. By summarizing these advances, this review aims to provide a clear framework for designing the next generation of antibody–drug conjugates and to support future research across oncology and beyond.

## 1. Introduction

Antibody–drug conjugates (ADCs) have evolved from early proof of concept to constructs that have established themselves into a sophisticated and significant therapeutic paradigm, especially in the field of oncology. Their advancement is indicative of a major change in drug design, from broadly cytotoxic agents to rationally engineered biologics that combines antigen selectivity, controlled intracellular drug release, and optimized and improved pharmacokinetics [1].

First-generation ADC efficiency was hindered by heterogeneous conjugation, unstable linkers, and a limited range of payloads, which hindered the effectiveness and increase in off-target toxicity. In contrast, advancements in technologies and research have led to an integrated design paradigm for modern ADCs where antigen biology, linker chemistry, payload potency, antibody format, and conjugation sites are considered collectively rather than individually. An integrated design strategy means that the antigen, antibody, linker, payload, and conjugation method are selected to work together in a biologically and chemically compatible way. ADC development now follows a systemic approach; each design choice is made with the others in mind to maximize efficacy, improve stability, and reduce toxicity. An example is the integrated design of trastuzumab deruxtecan, where HER2 targeting a stable but cleavable linker, a membrane-permeable topoisomerase I payload, and a high yet controlled drug-to-antibody ratio are combined to produce strong antitumor activity. This progress has been crucial in ADC growth from a narrow spectrum of hematologic applications to an expanding spectrum of combination therapies and solid tumor therapy [2].

The ADC field is currently evolving significantly in the nexus of chemistry, molecular biology, pharmacology, and computational sciences, which serves as the scientific justification for the present review. While existing reviews broadly capture separate aspects of computational modeling or clinical trials, a critical gap remains in synthesizing how data-driven architectures translate directly to real-world toxicity mitigation. This review specifically bridges that gap by synthesizing the literature from recent years into a functional taxonomy and workflow mapping structural design parameters to clinical outlays. Recent medicines have demonstrated that payload diversification and site-specific conjugation may additionally enhance efficacy and tolerability. In fact, clinically successful ADCs indicate that enhanced selection of targets and linker stability can translate into significant therapeutic benefits [3]. New hurdles have developed by the complex nature of these structures such as antigen heterogeneity, resistance mechanisms, interstitial pulmonary disease, and eye injury, along with manufacturing problems related to the product consistency.

These advancements are significant for scientific readers given that ADCs, as the name suggests, are no longer best understood as a single drug class characterized solely by cytotoxic delivery. Rather, they symbolize a platform technology wherein ongoing advancement relies on improved predictive development pipelines and deeper mechanistic knowledge [4]. Hand-in-hand with foundational principles established over the last decade—such as verifying high target expression levels, strong internalization rates, and minimal expression on normal tissues to isolate tumor-associated signals—the field of antibody engineering has rapidly progressed. The most significant point from previous years is that identifying a specific antigen involves more than just finding tumor-associated signals. It also entails choosing biologically appropriate target antigens having a high expression level, strong internalization, and minimal expression on normal tissues. These attributes assess whether an ADC has scope to minimize on-target/off-tumor adverse effects while delivering the payload effectively to tumor cells [5]. Concurrently, the field of antibody engineering has progressed. Bispecific (engineered to target two entirely distinct surface antigens (e.g., co-targeting HER2 and TROP2) to overcome tumor heterogeneity and antigen escape) and biparatopic (bpAbs) (engineered to simultaneously bind to two distinct, non-overlapping epitopes on the same target antigen (e.g., targeting two separate domains of the HER2 receptor) drastically enhancing binding avidity and receptor internalization velocities) antibody formats strengthen binding avidity, and help to address heterogeneous antigen expression, while Fc-engineered and glyco-engineered antibodies can effect performance and reduce non-specific interactions. In solid tumors, the major challenge is tumor tissue penetration. However, emerging ADC formats such as antibody fragments and nanobody-based formats may further improve tumor penetration and modify pharmacokinetic properties [6]. However, these alternative formats introduce distinct, non-trivial clinical and structural tradeoffs. Fragment and nanobody-based constructs often suffer from compromised pharmacokinetic profiles and abbreviated half-lives due to rapid renal clearance, while bispecific architectures and dual-payload strategies drastically elevate CMC (chemistry, manufacturing, and control) complexity, heighten immunogenicity risks, and introduce severe regulatory hurdles during multi-component clinical validation.

Moreover, significant advancements have also been made in payload and linker design chemistry. Cleavable linkers can support tumor or lysosome-specific release via protease, pH, or redox-sensitive mechanisms, whereas non-cleavable linkers can improve plasma stability and reduce premature payload release [7]. Beyond traditional microtubule inhibitors, payload innovation has now expanded to include topoisomerase I inhibitors, drugs that damage DNA, and early-stage non-cytotoxic payloads comprising immune modulators and degraders. The potential to combat intratumoral heterogeneity as well as resistance while maintaining controllable systemic toxicities renders dual-payload and tunable-payload approaches more attractive. Overall, these developments demonstrate the reasons why newly developed ADCs have produced a broader therapeutic impact than their predecessors [8].

Conjugation chemistry is another important domain of development. Although conventional lysine and cysteine conjugation techniques are frequently utilized, they often result in heterogeneous combinations exhibiting variable drug-to-antibody ratios (DARs) and concerns associated with hydrophobicity [9]. Conversely, site-specific conjugation technologies like engineered cysteines, enzymatic conjugation, glycan modification, and tag-based methods provide increased control over the conjugation site and DARs, which potentially improves stability, pharmacokinetics, and therapeutic index. All these developments are significant for improving the durability and consistency of ADCs, qualities that are becoming increasingly crucial [10].

Therefore, the purpose of this review article is to enhance the knowledge beyond our previously published review [11], particularly focusing on ADC research published primarily from 2019 to 2026. Its emphasis is on current applications of artificial intelligence (AI), AI- and data-driven target discovery, antibody engineering, linker and payload optimization, site-specific conjugation, clinical translation, toxicity management, and mechanisms of resistance. These approaches may encourage rational patient selection and dosing strategies, expedite the discovery process, and minimize late-stage attrition. In contrast to a purely descriptive summary, the present article is intended to provide a design rule framework for next-generation ADCs by integrating antigen biology, molecular architecture, developability, computational prediction, and translational considerations. By linking these elements, the review aims to offer a practical and conceptually organized basis for rational ADC design and future clinical implementation.

## 2. Antigen Selection and Antibody Engineering in Next-Generation ADCs

### 2.1. Refining Target Biology

Target selection has evolved from identifying highly expressed tumor antigens to refining target biology to maximize therapeutic index. While early ADC development largely prioritized target abundance, clinical experience has demonstrated that antigen density alone is insufficient to predict efficacy. Instead, successful antigen targets exhibit a combination of tumor-selective expression, efficient internalization, limited shedding, and sustained surface availability, all of which influence intracellular payload delivery and antitumor activity [12,13]. Beck et al. [12] emphasized that optimal ADC targets should display high expression on malignant cells with minimal expression in normal tissues to reduce on-target/off-tumor toxicity, while also undergoing rapid receptor-mediated endocytosis to facilitate lysosomal payload release. More recent analyses [13] have further highlighted the importance of addressing biological barriers such as intratumoral heterogeneity, dynamic antigen expression, and poor accessibility within solid tumors, which can limit uniform drug delivery despite high target expression. Notably, the clinical success of trastuzumab deruxtecan (T-DXd) in HER2-low breast cancer challenged the traditional assumption that only highly overexpressed targets are suitable for ADC therapy. Its efficacy in tumors with relatively low HER2 expression demonstrated that factors such as efficient internalization, a membrane-permeable payload, and a robust bystander effect can compensate for lower antigen density, thereby expanding the pool of clinically actionable targets [13,14,15].

**Figure 1:**
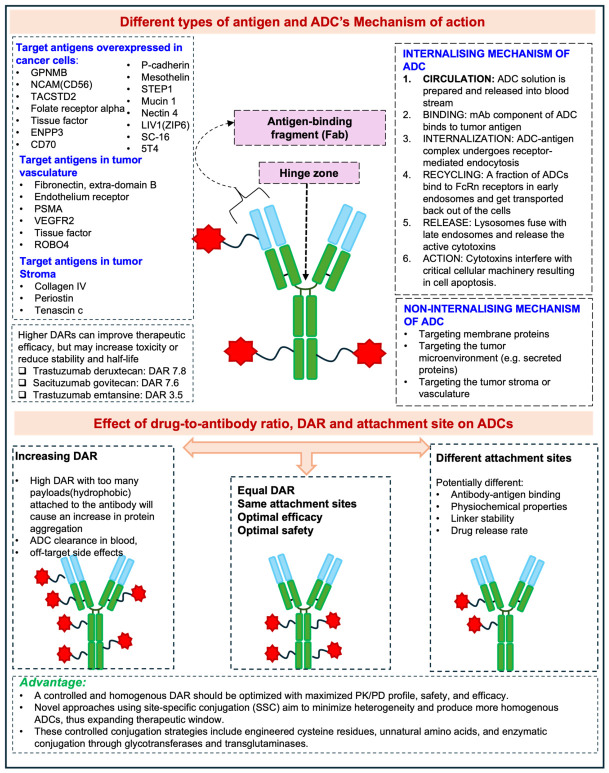
The figure illustrates different types of tumor antigens and ADC mechanisms of action, including comparative analysis of internalizing and non-internalizing pathways and relationship between drug-to-antibody ratio (DAR), physicochemical properties, and ADC efficacy.

These target biology considerations also help explain differences in ADC performance across disease settings. Hematologic malignancies have historically been more amenable to ADC therapy because targets such as CD19, CD22, CD33, CD79b, and BCMA are readily accessible in circulation and often exhibit more homogeneous expression patterns, facilitating efficient target engagement and payload delivery [12]. In contrast, solid tumors frequently present dense stromal barriers, elevated interstitial pressure, and heterogeneous antigen expression that limit ADC penetration and contribute to incomplete tumor coverage [13]. Furthermore, many solid tumor targets are tumor-associated rather than tumor-specific, which increases the risk of on-target/off-tumor toxicity and reduces the therapeutic window of conventional cytotoxic ADCs. Consequently, refining target biology increasingly involves evaluating not only antigen expression but also the biological context of the tumor microenvironment. Accordingly, conventional ADCs may be preferred in tumors with high antigen density, efficient internalization, and dependence on direct cytotoxic killing, whereas immune-stimulating antibody conjugates (ISACs) may be advantageous in tumors characterized by immune suppression, immune exclusion, or resistance to checkpoint blockade. This concept is demonstrated by Y101S, an ISAC developed by Li et al. that is derived from the TGF-β/PD-L1 bispecific antibody YM101 and engineered to activate the STING pathway, thereby promoting innate and adaptive antitumor immunity while reducing inflammatory off-target effects [16,17]. This approach highlights an important design principle for next-generation conjugates: when genomic, transcriptomic, or spatial profiling reveals enrichment of innate immune response pathways, immune exclusion, or mechanisms associated with immunotherapy resistance, immune-activating conjugates may offer advantages over conventional ADCs that rely primarily on direct tumor cell killing. Thus, target refinement is evolving beyond identifying surface antigens toward selecting therapeutic modalities that align with the underlying biology of both the tumor cells and their microenvironment, enabling more precise matching of ADC and ISAC platforms to specific resistance mechanisms and disease states. Examples of antigen classes and corresponding ADC mechanisms informed by these biological considerations are summarized in Figure 1.

### 2.2. Antibody Format Innovation

Antibodies are composed of different antibody binding fragments: Fabs and an Fc fragment. The former is involved in antigen recognition, and the latter is instrumental in recognition of a variety of Fc receptors which contribute to bioavailability regulation [12,18]. These receptors, therefore, are critical in immune modulation and carefully selecting or engineering the Fc portion will result in improved ADCs. The first consideration in antibody selection is the antibody subclass. There are four different subclasses related to the type of immunoglobulin: IgG1, IgG2, IgG3, and IgG4. The differentiating factor in ADC design consideration is serum stability, with IgG1 obtaining the longest stability of 21 days [18]. IgG1 additionally displays higher affinity for activating FcƴR than IgG2 and IgG4. As a result, IgG1 is used to activate receptors and IgG2 and IgG4 are used to deactivate the immune system. IgG1 remains the most selected type of immunoglobulin as an ADC backbone. This is supported by the clinical success of trastuzumab deruxtecan (T-DXd), which has significantly improved survival outcomes in HER2-positive and HER2-low breast cancer [14,15]. However, as discussed in Section 5.2, clinical toxicities including interstitial lung disease highlight the need to balance potent antitumor activity with safety considerations [19]. IgG2 and IgG4 backbones have limited clinical success. IgG2-related designs are beneficial when the therapeutic mechanism relies primarily on antigen-mediated internalization and payload delivery. Recently, the c-MET-directed ADC SHR-1826 has entered phase 1 clinical trials [20]. However, the efficacy data remains to be seen. Adoption of IgG2 ADCs for future development remains limited due to aggregation of IgG2 molecules and greater conjugation heterogeneity, both of which create manufacturing and developability challenges [3]. Additionally, IgG4 designs reflect strategies that prioritize targeted payload delivery over Antibody-Dependent Cellular Cytotoxicity (ADCC)-driven antitumor effects. This is the case for inotuzumab ozogamicin [21]. As previously mentioned, Fc engineering is another strategy to improve ADC properties. Typically, this refers to antibody glycoengineering. One common method is to reduce the amount of fucose in the Fc regions [12]. Increased affinity for FcƴRIIIa is observed due to increased ADCC activity. Blenrep, a B-cell maturation antigen (BCMA)-directed antibody, is a well-known example of Fc engineering. It has been recently reapproved in October 2025 in combination with bortezomib and dexamethasone for relapsed refractory multiple myeloma [22].

Bispecific antibodies are an emerging strategy to increase efficacy of ADCs by binding to two different antigens [23]. Bispecific antibodies are classified into two categories: IgG-based and fragment-based. IgG-based antibodies contain an antigen recognition site and an Fc fragment similar to native antibodies. In recent years, there have been significant breakthroughs in IgG-based bispecific antibodies. New strategies have emerged in the treatment of colorectal cancer which remains an increasing concern due to rising incidence rates among young adults [24]. Specifically, one strategy is conjugating a ferroptosis inducer, which is responsible for cell death through iron-dependent lipid peroxidation, to cell adhesion molecule CDH17 and transmembrane receptor GUCY2C that are overexpressed in colorectal cancer [25]. This study highlighted and implemented several important design considerations for next-generation bispecific ADCs. Specifically, antibody valency and binding-site architecture were shown to influence target engagement and internalization. The authors demonstrated that an optimized 2 + 2 bispecific format enhances antibody binding strength and payload delivery while minimizing steric hindrance. Overall, this demonstrates that future bispecific ADC development will require careful antigen selection as well as rational optimization of antibody geometry to maximize efficacy and minimize off-target toxicity. IgG bispecific antibodies that are currently in phase III clinical trials can be found in Table 1.

### 2.3. Emerging Formats

Nanobodies can provide an alternative scaffold compared to monoclonal antibodies and bispecific antibodies because of their small size, high stability, and ability to recognize epitopes that may be less accessible to conventional IgG formats. Their low molecular weight can improve tissue penetration and may support more homogenous tumor distribution. Additionally, through genetic engineering, nanobodies have a higher expression yield in bacterial systems, which can simplify the manufacturing process [69]. However, these same properties can be a double-edged sword and present design liabilities for ADC development. Unmodified nanobodies demonstrate rapid clearance, resulting in short circulation times that may limit tumor exposure and intracellular payload delivery [69]. To counteract this issue, nanobody-based therapeutics are investigating half-life extension strategies. These include engineering methods targeting albumin binding domains [69,70,71], fc fusions, PEGylation constructs [69,72] or multivalent constructs [69,73]. One issue with these methods is that the risk of diminishing tissue penetration increases. These tradeoffs are illustrated in clinical nanobody therapeutics. Caplacizumab, the first FDA-approved nanobody therapeutic, demonstrated clinical efficacy in acquired thrombotic thrombocytopenic purpura but also highlighted the need to monitor anti-drug antibody formation, with treatment-emergent antibodies detected in 3% of patients in the HERCULES trial [74]. Conversely, Ozoralizumab was specifically engineered with a human serum albumin binding domain to overcome rapid clearance, extending its plasma half-life to approximately 18 days and enabling once-monthly dosing in patients with rheumatoid arthritis [75]. Therefore, successful nanobody–drug conjugates will require careful optimization of tissue penetration, circulation half-life, renal exposure, payload potency, target-mediated internalization, and other factors rather than relying solely on the intrinsic advantages of the nanobody scaffold.

## 3. Linker and Payload Innovation to Optimize Therapeutic Index

**Figure 2:**
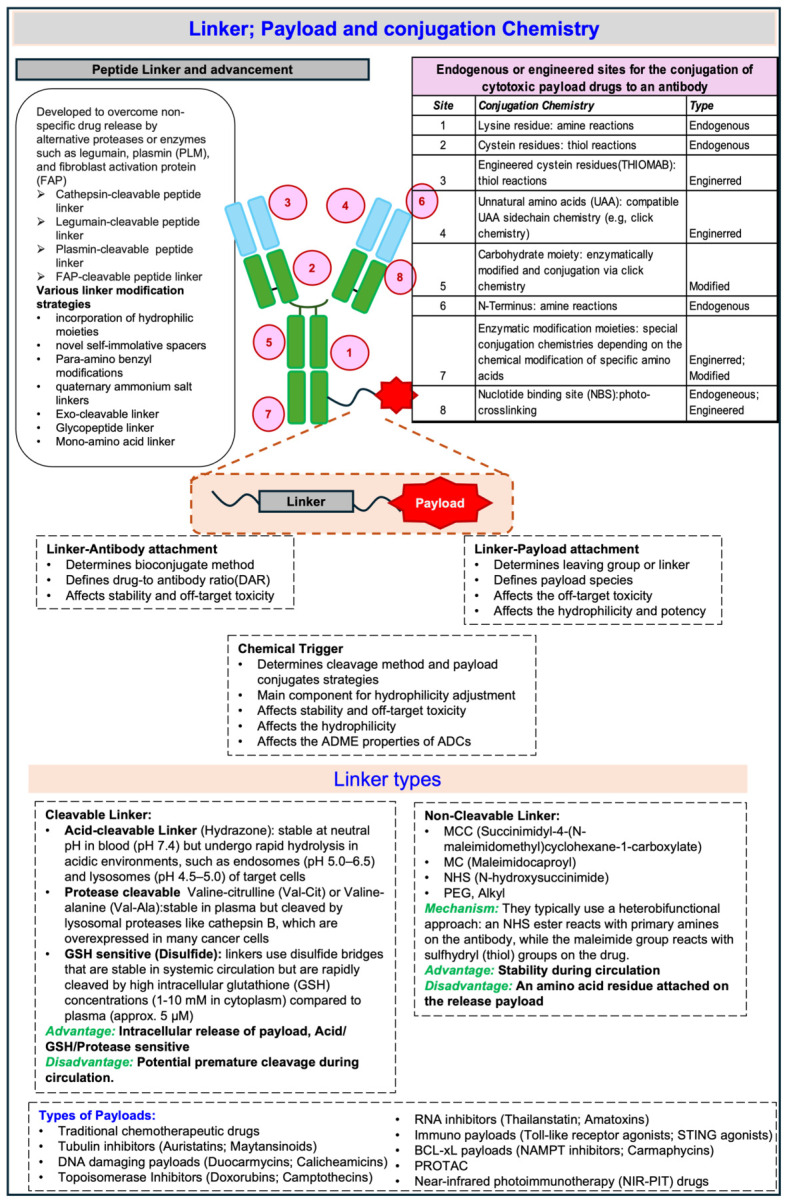
Overview of ADC components: linkers, payloads, and conjugation chemistry.

The therapeutic index of an ADC is determined not by its cytotoxic payload alone, but by the precision with which that payload is released within the tumor microenvironment and withheld from healthy tissue. Conventional ADCs were constrained by binary linker logic, making them stable in blood circulation and liable at the target. This strategy has enabled the development of several clinically successful ADCs. However, limitations remain due to factors such as intratumoral heterogeneity, variable antigen expression, differences in intracellular processing, and the pharmacokinetic complexity of systemic distribution. In recent years, the ADC field has made significant progress through the incorporation of combinatorial conjugation techniques, payload class diversification, and innovation in linker chemistry (Figure 1) [76,77]. These advances are intended to address biological barriers such as intratumoral heterogeneity and may improve the therapeutic window in selected disease settings. Therefore, ADC development is increasingly moving beyond optimization of individual components towards consideration of how target biology, linker stability, payload mechanism, and bystander activity collectively influence therapeutic performance. This integrated strategy has emerged as an important design consideration for next-generation ADCs. However, the optimal combination of these features remains dependent on the biological context of each disease indication. The functional and structural core of ADC design continues to be the linker. Cleavable linkers are designed to exploit physicochemical differences between the tumor microenvironment and systemic circulation, such as, lysosomal acidification (pH 4.5–5.0), enzymatically liable linkages, heightened cathepsin B and cathepsin L protease activity, and elevated intracellular glutathione levels in solid tumors, [78,79]. Disulfide-based systems, hydrazone bonds, and valine–citrulline dipeptide (Val-Cit) linkers are designed to leverage at least one of these gradients, resulting in varying selectivity and release kinetics. One important consequence of many cleavable linker designs is the potential for a controlled bystander effect. Free payload can diffuse across cell membranes to eradicate nearby antigen-negative tumor cells. This attribute is clearly advantageous in heterogeneous tumors but is potentially harmful in normal tissues exhibiting low antigen expression [78,80] (Figure 2).

Succinimidyl-4-(N-maleimidomethyl) cyclohexane-1-carboxylate (SMCC) is an example of non-cleavable linkers used in the synthesis of ado-trastuzumab emtansine (T-DM1), which results in charged, membrane-impermeant catabolites that limit payload action to antigen-positive cells. This leads to a decrease in off-target diffusion. However, it negatively impacts bystander coverage [81]. The clinical differences between T-DM1 and trastuzumab deruxtecan (T-DXd), which incorporates a topoisomerase I inhibitor payload with a strong bystander effect and a tetrapeptide-based cleavable linker, demonstrate how linker selection directly contributes to varying levels of therapeutic efficacy across different expressions of the HER2 gene [14]. The clinical differences between T-DM1 and T-DXd illustrate an important design tradeoff in linker selection. A strong bystander effect may be advantageous in tumors with heterogeneous antigen expression because payload diffusion can eliminate neighboring antigen-negative cells that would otherwise escape treatment. However, increased payload permeability may also increase exposure of normal tissues to cytotoxic metabolites, potentially narrowing the therapeutic window. For example, the superior efficacy of T-DXd in HER2-low tumors has been partially attributed to its membrane-permeable payload and bystander activity, although the relationship between enhanced payload diffusion and toxicities such as interstitial lung disease remains an active area of investigation [14,19]. Therefore, the optimal degree of bystander activity is likely to vary according to tumor biology rather than being universally beneficial.

Diversification of payloads has significantly expanded the therapeutic potential of ADCs. First-generation ADC payloads such as maytansinoids and auristatins, which function as microtubule inhibitors, established clinical proof of concept. However, these payloads are associated with several limitations, including elevated susceptibility to multi-drug resistance through P-glycoprotein-mediated efflux, limited activity in non-proliferating cells, and relatively narrow therapeutic windows [82]. Consequently, recent ADC development has increasingly incorporated camptothecin-derived DNA topoisomerase I inhibitors such as the DXd payload incorporated into trastuzumab deruxtecan and Datopotamab deruxtecan [83].

These payloads maintain their function against MDR-phenotype cells, exhibit membrane permeability that contributes to bystander killing, and demonstrate strong cytotoxicity at sub-nanomolar concentrations. These characteristics have contributed to improved clinical efficacy in several heavily pretreated solid tumor populations [84]. However, increased membrane permeability and bystander activity may also contribute to overall toxicity. For example, trastuzumab deruxtecan and Datopotamab deruxtecan have been associated with interstitial lung disease (ILD) and pneumonitis, highlighting the importance of balancing efficacy with safety during payload selection. DNA-damaging payloads, including duocarmycin derivatives and pyrrolobenzodiazepine (PBD) dimers, represent another highly potent payload class. Although these agents demonstrate picomolar cytotoxicity, their clinical development has been limited by narrow therapeutic windows and significant systemic toxicities, including hematologic and pulmonary adverse events [76]. Several PBD-containing ADCs demonstrated promising preclinical activity but experienced safety-related challenges during clinical development, suggesting that increased payload potency alone is insufficient to ensure clinical success.

In addition to conventional cytotoxic payloads, a paradigm change in favor of non-cytotoxic payloads is currently taking place. Proteolysis-targeting chimeras (PROTACs), stimulator of interferon genes (STING) pathway activators, and toll-like receptor agonists are currently being attached to antibodies with the goal of inducing targeted protein degradation or reprogramming the tumor microenvironment, respectively, in lieu of the systemic immunosuppression that results with traditional cytotoxic payloads [85].

The development of ADC architecture that may concurrently handle intratumoral antigen heterogeneity and acquired resistance without proportionately increasing systemic toxicity is perhaps the field’s most critical clinical concern. In preclinical models, dual-payload ADCs combine two different cytotoxic agents on a single antibody scaffold, which shows synergistic antitumor activity and is less likely to develop resistance in comparison to monotherapy [86].

Ren et al. have investigated a novel dual-payload ADC platform that combines Exatecan (topoisomerase I inhibitor) and Triptolide (RNA polymerase II inhibitor) to improve antitumor efficacy and overcome resistance in cancer patients [87]. Through site-specific conjugation using sortase-mediated ligation (SML), the authors achieved precise stoichiometric control for dual-payload loading. SML is a specific, enzymatic protein engineering method that links antibodies with site-specific proteins, peptides, or synthetic molecules through recognition of the LPETG motif by sortase A (often from *S. aureus*) [88]. This has drastically improved heterogenous DAR to uniform distribution. However, dual-payload ADCs also introduce additional challenges in manufacturing and characterization. Precise control of payload stoichiometry, conjugation site occupancy, and DAR distribution must be maintained throughout production, while the presence of two distinct payloads increases the complexity of analytical characterization and batch-to-batch consistency assessments [87,89]. Additionally, tunable-payload strategies, in which the ratio of two payloads is adjusted to address specific resistance mechanisms, may complicate process standardization and quality control because different payload combinations could require independent optimization and validation [89]. Therefore, although dual-payload and tunable-payload ADCs have demonstrated promising preclinical activity against tumor heterogeneity and resistance, their translation into large-scale clinical manufacturing and regulatory development remains to be established [89].

Critical gaps continue to exist at multiple levels. The degree to which site-of-release fidelity is compromised by cathepsin expression heterogeneity within a tumor mass has not been systematically measured clinically, and the factors influencing differential linker cleavage efficiency across tumor types and individual patients are not fully understood. Despite its conceptual appeal, the immunomodulatory payload domain lacks confirmed pharmacodynamic biomarkers which would make it possible for logical selection of patients. Furthermore, the long-term effects of chronic low-level bystander cytotoxicity on tumor-adjacent healthy tissues remain poorly understood, particularly in visceral organs and the central nervous system. Addressing these questions will likely require integrated preclinical and clinical platforms that combine longitudinal PK/PD modeling with spatially resolved tumor profiling. Emerging data-driven approaches, including machine learning models trained on multi-omics, imaging, and clinical datasets, may further improve prediction of linker cleavage, payload distribution, treatment response, and toxicity across diverse patient populations. Therefore, integrating computational modeling with experimental and clinical data may help transform linker-payload optimization from a largely empirical process into a more predictive and biologically informed design strategy.

## 4. Conjugation Technologies, DAR Control, and Developability

### 4.1. Conventional Lysine/Cysteine Conjugation

**Figure 3:**
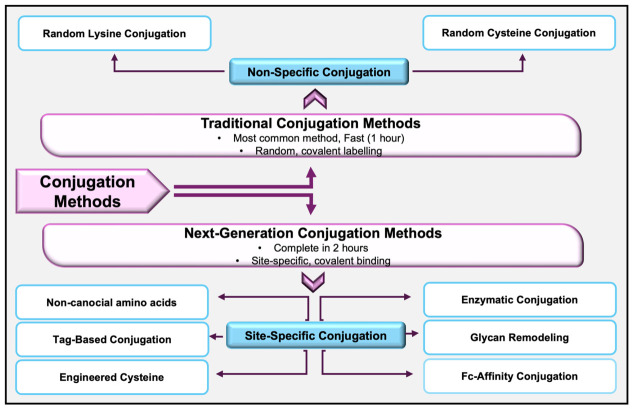
Evolution of ADC conjugation strategies. From traditional to site-specific methods. Traditional methods typically focus on stochastic conjugation to lysine or cysteine residues, often resulting in heterogeneous mixtures. Next-generation methods (site-specific) utilize technologies such as enzymatic conjugation, unnatural amino acid incorporation, or engineered cysteine residues to achieve precise drug-to-antibody ratios (DARs) and improved homogeneity.

Traditionally, ADC conjugation has been accomplished through various lysine and cysteine residues on the antibody (Figure 3). Lysine residues contain highly nucleophilic NH_2_ groups at neutral pH solution allowing for easy attachment via SN2 reactions. Because there are many solvent-exposed lysine residues, selectivity remains challenging. This lack of selectivity has a wide range of adverse effects including reduced efficacy and toxicity concerns. The mechanism of cysteine conjugation involves the reduction in interchain disulfides bonds to expose free thiol groups [9]. Cysteine conjugation is more selective than lysine conjugation, and as a result, heterogeneity is decreased, typically yielding a DAR of approximately 2 to 8 payloads [90]. Among conjugation strategies for cysteine residues, maleimide conjugation remains the preferred choice due to high yields and favorable conditions [9]. However, there are certain drawbacks of using this moiety, namely retro-Michael instability which can reduce safety of the ADC [91]. Further chemical modifications have been investigated to negate these drawbacks but are beyond the scope of this review [92,93,94,95].

### 4.2. Site-Specific Conjugation Platforms

Recent trends and methods have been evolving to site-specific conjugation platforms for lysine and cysteine residues (Figure 3). One specific method is template-directed and has been used extensively to target lysine residues [96]. The templates use Fc-binding peptide or affinity ligands which allow positioning and non-covalent binding of moieties to specific lysine residues. The most targeted lysine residues are K248 and K288 [97,98,99], which provide specific structural advantages by preserving antigen binding due to their location in the Fc domain. Additionally, K248 conjugation demonstrated a wider therapeutic index compared to FDA-approved Kadcyla, and overall, template-directed methods targeting these lysine residues maintain homogenous DARs, resulting in better PK/PD properties [99]. Cysteine residues have also been subjected to site-specific conjugation. This involves engineering of an amino acid to a cysteine by means of site-directed mutagenesis or vector insertion into the light or heavy chain sequence of an antibody [90]. The most common technology is known as THIOMAB [100]. Site selection is based on numerous factors [92] and often brings about issues regarding ADC preparation. For example, the free thiol is exposed from engineered cysteines via partial reduction and reoxidation. This can lead to overoxidation, and improper protein folding caused by reforming disulfide bonds. However, Liao et al. report a one-pot synthesis strategy for negating these unintended consequences that is used to produce two cysteine mutant antibodies in the light or heavy antibody chain (LC214 and HC220). This technology is just one example of improving homogenous DARs and the therapeutic index [101].

Although site-specific conjugation improves control of DAR and reduces product heterogeneity, it remains unclear how much these improvements contribute to clinical outcomes. Several approved ADCs, including trastuzumab emtansine, trastuzumab deruxtecan, and sacituzumab govitecan, utilize conventional conjugation strategies and have demonstrated significant clinical benefit [15,82,102,103,104]. These examples suggest that homogenous DAR distributions alone are insufficient to determine ADC efficacy. Instead, ADC performance is influenced by multiple factors including target selection, payload potency, linker stability, pharmacokinetics, and tumor biology [105]. Therefore, the advantages of site-specific conjugation must be weighed against the additional manufacturing and analytical complexity required to produce these constructs. Site-specific approaches may be most beneficial for ADCs containing highly potent payloads or those with narrow therapeutic windows where tighter control of drug loading could improve the therapeutic index. While site-specific conjugation consistently improves product quality attributes [96,100,106], additional clinical experience is needed to determine whether these improvements translate into meaningful therapeutic advantages over optimized stochastic conjugation strategies.

### 4.3. Developability Considerations

ADC design challenges also extend beyond target selection and conjugation into developability and formulation. Unlike unconjugated antibodies, the developability of ADCs is influenced by the combined properties of the antibody, linker, payload, conjugation site, and DAR [107]. Homogeneity is needed for ADCs to show improved stability and better formulation. Therefore, homogeneity should not only be considered in terms of antibody purity, but also the distribution of DAR species, unconjugated antibody, free payload, and batch-to-batch reproducibility [107]. Heterogeneous DAR distributions can complicate analytical characterization and generate ADC populations with different pharmacokinetic, efficacy, and safety profiles.

Stability and solubility are closely related considerations because conjugation often introduces hydrophobic linker-payload structures. Increased hydrophobicity has been associated with protein aggregation, reduced solubility, and decreased conformational stability. For example, Hobson et al., [108] demonstrate that optimizing linker-payload chemistry directly impacts subcutaneous dosing by altering solution stability (including viscosity, solubility, melting temperature, hydrophobicity). Likewise, the incorporation of hydrophilic linker elements such as PEG or sulfonate groups can improve solubility and pharmacokinetic properties [109]. However, these modifications may also influence payload release, potency, and manufacturability.

Formulation presents additional challenges for ADC development. Many approved ADCs are supplied as lyophilized products because liquid formulations may be susceptible to aggregation, payload degradation, and long-term stability issues. Additionally, stability following dilution must be evaluated because adsorption losses and particle formation can affect product quality, particularly at low concentrations [110]. These challenges become increasingly important for high-concentration and subcutaneous formulations where viscosity and solubility may limit product development.

## 5. Clinical Translation, Toxicity Management, and Resistance

### 5.1. Evolving Clinical Landscape

Recent regulatory approval by the FDA and EMA have expanded the utilization of ADCs in the oncology field including both hematologic malignancies and solid tumors, transitioning and upgrading them from niche salvage therapies to critical treatments [3]. However, this clinical expansion is marked by significant heterogeneity; therapeutic benefit varies drastically based on target antigen density, tumor microenvironment factors, and intrinsic resistance mechanisms across different tumor types and lines of therapy. Furthermore, widespread clinical integration faces severe global access disparities driven by high manufacturing complexity and prohibitive treatment costs, frequently confining these therapeutics to resource-rich settings. Earlier ADCs targeting CD33, CD22, CD79b, BCMA, and CD19 are efficacious, and they are continuing to show success in blood cancers. Newer ADC constructs against HER2, TROP2, Nectin-4, FRα, and emerging antigens like B7-H3 and CDH6 have demonstrated improved efficacy in breast, lung, urothelial, ovarian, and other epithelial cancers, even amid antigen heterogeneity [111,112,113]. For instance, Sacituzumab govitecan (IMMU-132) is reshaping treatment algorithms for metastatic triple-negative breast cancer, while trastuzumab deruxtecan (T-DXd; DS-8201) has moved into HER2-low breast cancer maintenance post-neoadjuvant therapy. This expansion aligns with critical changes toward frontline and adjuvant settings [15,102]. T-DXd comprises a tumor-selective cleavable tetrapeptide linker that binds trastuzumab to deruxtecan, a strong topoisomerase-I inhibitor payload. This payload has a high DAR = 8, is membrane-permeable, and has strong negative effects on nearby heterogeneous or HER2-low cancer cells. The key DESTINY-Breast03 trial indicated that T-DXd is significantly superior to T-DM1 for patients with prior therapy for HER2-positive metastatic breast cancer, with a median survival rate of 28.8 months in comparison to 6.8 in T-DM1. This remarkable development has once again lifted the benchmark for HER2-targeting second-line treatment [103]. Despite raising this benchmark, T-DXd’s real-world deployment requires strict vigilance due to severe class-related toxicities. In particular, the risk of drug-induced interstitial lung disease (ILD)/pneumonitis—which can be fatal if unrecognized—and high hematologic toxicity (e.g., neutropenia) substantially shape its clinical monitoring protocols, necessitating routine CT surveillance and proactive dose modifications. Similarly, sacituzumab govitecan incorporates a hydrolysable cleavable linker that connects a humanized anti-TROP-2 monoclonal antibody to the topoisomerase-I inhibitor SN-38 (the active metabolite of irinotecan).

A growing body of research demonstrates that evaluating these agents within combination treatment regimens will further accelerate this evolution. Specifically, trials for HER2-positive and other solid tumors are investigating combination therapies that pair ADCs with PARP inhibitors, immune checkpoint inhibitors, or chemotherapeutic agents. Notably, ADCs such as Enfortumab vedotin (anti-Nectin-4) have been combined with anti-PD-L1 (Atezolizumab) and anti-PD-1 (Pembrolizumab) antibodies, while regimens involving T-DM1 (trastuzumab emtansine) with anti-CTLA-4 (Ipilimumab) or with paclitaxel are being explored to enhance therapeutic response and overcome resistance to immunotherapy in solid tumors. A potential treatment option for patients with metastatic or resistant TNBC is sacituzumab govitecan with cisplatin, which has been proven in clinical trials to dramatically enhance ORR and PFS [104]. Mechanistically, these combinations leverage biological synergy: pairing ADCs with checkpoint inhibitors utilizes payload-induced immunogenic cell death (ICD) to recruit T-cells into the tumor microenvironment (turning “cold” tumors “hot”), while combinations with PARP inhibitors yield catastrophic DNA double-strand breaks when coupled with topoisomerase or alkylating payloads. However, key translational questions remain as to which combinations are most promising. Current biomarker strategies remain limited to baseline antigen expression rather than dynamic microenvironmental indicators. Furthermore, these regimens face major design and clinical hurdles, including overlapping hematologic or gastrointestinal toxicities, complex sequencing dilemmas (concurrent vs. sequential deployment post-resistance), and compounded financial strain. As a result of these advancements, ADCs are now positioned as platform therapeutics compared to line-specific interventions, and ongoing studies are examining their potential in perioperative and minimal residual disease settings.

### 5.2. Toxicity Framework

ADC toxicities require structured treatment leveraging the on-target, off-tumor versus off-target dichotomy, despite being better than first-generation designs of ADCs. Hepatotoxicity driven by calicheamicin catabolites in gemtuzumab ozogamicin is an example of an off-target event. Conversely, on-target, off-tumor effects result from physiologic antigen expression, such as TROP2 in lung and intestinal epithelia, causing gastrointestinal distress with sacituzumab govitecan [114]. Across all ADC classes, one of the common adverse effects is an increase in hepatic enzyme, which is often considered for prompting baseline liver-function assessment to monitor drug induced liver toxicity. Dose holds are generally advised for Grade 2 or higher elevations in alanine aminotransferase (ALT) or aspartate aminotransferase (AST), particularly when accompanied by elevated bilirubin [115]. Agents such as mirvetuximab soravtansine (MIRV), and belantamab mafodotin are associated with ocular adverse events, including keratopathy and dry eyes, which require routine slit-lamp exams and artificial tears. High-resolution CT surveillance and prompt steroid therapy are necessary for suspected cases of interstitial lung disease, a potentially lethal side effect of topoisomerase I inhibitor ADCs such as trastuzumab deruxtecan [19].

Hematological suppression, neutropenia, and thrombocytopenia are common and often dose-limiting adverse events associated with nearly all ADCs. The toxicity profile often relates directly to the payload component (e.g., MMAE, SN-38, Dxd) causing off-target effects or premature release. Effective management strategies, that are often implemented by multidisciplinary teams, have transformed these manageable AEs into controlled aspects of care, preventing treatment discontinuation and optimizing dose intensity. Myelosuppression affects nearly all ADCs and is mitigated through granulocyte colony-stimulating factor (G-CSF) prophylaxis, strict transfusion thresholds, and schedule extensions from a 3-week (Q3W) to a 4-week (Q4W) cycle, which provides more time for bone marrow recovery, reducing the severity of cumulative cytopenia. Evidence-based protocols incorporating risk-adapted dosing, multidisciplinary monitoring, and patient education have transformed manageable adverse events into controlled aspects of ADC therapy. To successfully transition from empirical management (such as dose holds and CT surveillance) to design-driven improvement, clinical toxicities must be systematically mapped directly back to architectural design choices (linker stability, payload properties, and DAR configurations) (Table 2).

### 5.3. Mechanisms of Resistance and Current Solutions

**Figure 4:**
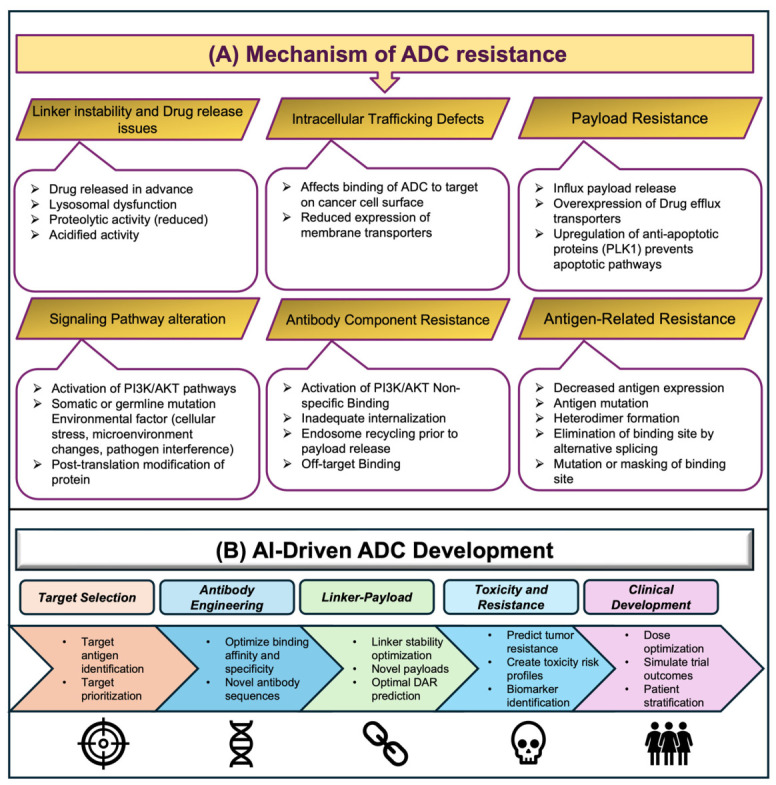
(**A**) Multidimensional mechanisms of tumor resistance to antibody–drug conjugates. (**B**) Integrating artificial intelligence (AI) into the ADC development cycle: from target identification to clinical success. AIs accelerate ADC development by predicting optimal targets and simulating complex molecular interactions between the antibody, linker, and payload. These models streamline design, improve therapeutic index, and identify resistance biomarkers to guide more precise clinical applications.

One of the major limitations of ADC-based therapy is the development of resistance [116], which can emerge at multiple points across the delivery cascade. First, antigen modification decreases its binding affinity, as observed in HER2-loss post-trastuzumab deruxtecan therapy. Second, trafficking defects that export intact ADCs extracellularly or trap them in non-lysosomal compartments and lysosomal hypoacidification hinder efficient payload release [117]. Third, an increase in efflux of membrane-permeable payloads via multi-drug resistance 1 (MDR1/ABC transporters) reduces cytotoxicity and leads to acquired resistance. In addition, resistance issues are exacerbated by tumor heterogeneity, where antigen-low clones predominate the tumor due to selection pressure (Figure 4A). To overcomes these issues, a few possible solutions have been utilized which includes development of bispecific ADCs that potentially targets dual epitopes (e.g., HER2/HER3) or antigens (e.g., EpCAM/CDH17), protect against single-target escape, and restore avidity and internalization, specially to antigen-low tumor areas [118]. A similar approach involves dual-payload designs that combines microtubule and topoisomerase inhibitors, which have demonstrated synergetic activity against heterogeneous xenografts and the ability to overcomes payload-specific resistance in preclinical models. Further, modulating the tumor microenvironment through combination therapies represents a highly promising avenue. In particular, PARP or EGFR inhibitors can limit DNA repair or circumvent signaling in sensitized tumors, whereas PD-1 inhibition may encourage T-cell infiltration after ADC-mediated payload release. Finally, sequential strategies such as ADC priming prior to CAR-T therapy help in improving outcomes by lowering antigen-dense tumor populations. Collectively, these strategies in combination increase ADC durability and efficacy in comparison to monotherapy [119,120].

To circumvent resistance mechanisms like antigen escape or efflux pump upregulation, several next-generation strategies are actively being pursued, though their developmental maturity varies significantly. (i) Clinically validated strategies: sequential ADC utilization (e.g., utilizing a Topo-I inhibitor ADC following a microtubule-inhibitor ADC) and targeting altered trafficking pathways have demonstrated real-world efficacy in breast and urothelial cohorts. (ii) Preclinical and early-phase concepts: bispecific ADCs (targeting two distinct antigens simultaneously to prevent antigen loss) and dual-payload ADCs (co-delivering synergistic payloads to prevent clonal resistance) remain largely speculative, currently confined to preclinical validation or early phase I trials.

The translation of these advanced formats is severely restricted by heightened manufacturing complexity, soaring development costs, and unpredictable, cumulative systemic toxicities arising from multi-payload or multi-target off-tumor interactions.

## 6. Artificial Intelligence and Computational Technologies in ADC Research

### 6.1. Target Discovery and Antibody Design

Artificial intelligence (AI) integrates multi-omics datasets including transcriptomics, proteomics, metabolomics, and digital pathology to identify ADC targets. By evaluating tumor-to-normal expression ratios, machine learning algorithms can rapidly screen for highly tumor-enriched, internalizing antigens [121]. For instance, Fang et al. developed in silico platforms to identify 75 surface proteins, by using an ADC target atlas that includes an algorithm which screens transcriptomic, proteomic, and genomic data against safety and internalization criteria across 19 solid tumor types [122]. Likewise, AI-driven platforms that use tissue microarray confirmation of computational prioritization scores have validated NECTIN4 and HER2 as high-priority targets in urothelial carcinoma [123]. Structure-based antibody engineering employs protein language models (DeepAb, Ig-VAE) and docking techniques to optimize paratope geometry, CDR-H3 loop diversity, and bispecific chain pairing for dual-targeting ADCs [124]. Quantifiably, these computational target discovery platforms have compressed the candidate antigen selection timeline from years to months, while protein language models routinely yield a 2- to 3-fold improvement in experimental screening hit rates compared to traditional empirical workflows, as validated by prospective internal screening metrics.

AI-based research, including colocalization and high-content imaging data analysis, enables the identification of promising internalizing antibodies that exhibit strong tumor killing activity in functional assays and has potential to develop into full-length IgGs. This information has shifted from empirical-based screening towards design–build–test–learn (DBTL) cycles [124,125]. Advancements in artificial intelligence (AI) employing the DBTL framework replace slow, empirical screening with predictive, iterative optimization, fundamentally transforming and accelerating ADC development. Moreover, it can optimize antibody affinity, predict linker stability, and screens potential payloads, thereby reducing development times and improving ADC therapeutic windows against various cancers (Figure 4B). Crucially, whereas traditional DBTL workflows apply these steps in a linear, stepwise fashion, AI provides a distinct framework by uniquely accelerating the “Learn-to-Design” transition. Rather than discarding failed candidate data, active learning loops ingest negative experimental assay results to dynamically map multi-parameter design rules, effectively preventing downstream developability bottlenecks in subsequent iterations.

### 6.2. In Silico Optimization of ADC Components

Machine learning models’ algorithms have helped in guiding cleavable vs. non-cleavable linker selection, predicted linker cleavage kinetics under serum condition, lysosomal environment, and tumor microenvironmental condition [80,124]. In addition, quantum chemical-informed neural networks (CNN)s and graph-based models can compute payload–antibody compatibility, conjugation site effects on binding affinity, and DAR-dependent hydrophobicity [121]. In HER2-targeted ADCs for breast cancer, deep learning models trained on antibody–antigen complex structure have been used to identify optimal site-specific cysteine conjugation sites. This computational approach ensures maximum receptor binding affinity while minimizing intracellular degradation and improving overall structural stability. Notably, these deep learning models are increasingly being used to computationally guide optimal site-specific conjugation chemistry. By predicting precise geometric coordinates, these models complement—rather than replace—established, traditional site-specific engineering platforms such as engineered cysteine ADCs or THIOMAB technology (a long-standing, non-AI platform). This hybrid computational–biochemical approach guarantees that a cytotoxic payload conjugates at predetermined, partially solvent-accessible locations, such as engineered cysteines (e.g., LC-V205C, HC-A114C) [9,126]. Further, Arsiwala et al. explained about Ginkgo’s PROPHET-Ab platform, which tests 246 antibodies (106 approved, 135 clinical-stage) across 10 developability assays (PI, aggregation, stability, expression, etc.). However, several critical constraints must be acknowledged within this “data-driven” design narrative. First, there is an acute scarcity of labeled, public, ADC-specific datasets, forcing models to rely heavily on general antibody sequences. Second, a severe domain shift exists between simplified in vitro high-throughput screening data and the highly complex physiological microenvironments of actual human patients. Finally, training highly parameterized algorithms on small, restricted datasets introduces a high risk of model overfitting, which can result in severe in vivo performance deviations during prospective preclinical validation. This platform was specifically designed to generate datasets for AI/ML model training, which enables data generation at the scale necessary to build more advanced and effective ML models to predict antibody developability [127]. These methods speed up optimization while preventing late-stage developability problems by reducing the design space from thousands of variants to dozens of high-confidence possibilities.

### 6.3. Model-Informed and AI-Assisted Clinical Development

Quantitative systems pharmacology (QSP) and physiologically based pharmacokinetic (PBPK) models predict ADC exposure–response relationships by integrating target expression, internalization rates, payload bystander effects, and organ-specific toxicities [128]. Additionally, a mechanistic minimal PBPK (mPBPK) model that compares payloads and bystander effects can accurately predict first-in-human dosing, as demonstrated for loncastuximab tesirine. However, while first-in-human scaling represents a well-validated application of PBPK modeling, other complex endpoints remain exploratory. For instance, using these models to predict risk-stratified ILD for DXd-based ADCs remains an aspirational framework under active optimization. Prior to clinical testing, virtual patient cohorts model combination synergies, dose–schedule tradeoffs, and adaptive trial designs [129]. In addition to this, AI decision-support technologies utilize data from the real world for predicting toxicity such as interstitial lung disease, ocular, hematologic, assist in risk-adapted regimens and biomarker-enriched enrollment [130]. While promising, these tools currently serve as methods for clinical hypothesis generation rather than an established standard of care. Reinforcement learning frameworks model tumor debulking and microenvironmental changes after payload release to optimize sequencing (ADC → CAR-T, ADC → PD1). These methods transformed empirical escalation into predictive clinical translation, supporting trastuzumab deruxtecan dose decisions, and are currently directing next-generation bispecific ADC combos [131].

## 7. Conclusions and Outlook

It is anticipated that fourth-generation ADCs would emerge using an integrative design methodology that optimizes conjugation technique, computational prediction, linker-payload chemistry, antigen biology, and antibody format concurrently instead of separately. Specifically, this fourth generation is defined by three distinct criteria that differentiate it from current clinical paradigms: (i) mandatory, structure-guided AI integration to predict multi-parameter developability, (ii) the routine integration of standardized PBPK/QSP pipelines to simulate human translational behavior before phase I trials, and (iii) a shifting dominance from traditional cytotoxic payloads to non-cytotoxic, targeted therapeutic modalities. The most promising ADCs are expected to combine tumor-specific antigen expression, efficient cellular internalization, and a payload release mechanism that maintains systemic stability despite facilitating intracellular activation. Additionally, AI-enabled assessments are being applied more often to evaluate pharmacokinetic conduct, model developability liabilities, and prioritize targets with the goal to reduce the time between concept to candidate selection. These strategies are particularly crucial for ADCs because slight modifications to the conjugation site, drug-to-antibody ratio, or payload hydrophobicity may significantly impact tolerance and efficiency [132]. However, current expectations must be carefully tempered: contemporary AI/ML models frequently struggle to accurately predict these non-linear, context-dependent physiological effects. Subtle structural shifts often generate profound, chaotic changes in systemic clearance and off-target toxicities that machine learning architectures cannot yet reliably anticipate, underscoring that computational tools must remain tightly tethered to rigorous empirical validation.

A major challenge lies in expansion of ADC concepts beyond classical cytotoxic payloads. Recent research shows that ISACs-ADCs and ADC-like conjugates delivering immunostimulatory molecules can improve antitumor immunity and combat resistance related to cytotoxic mechanisms [133]. Beyond oncology, ADCs are increasingly being investigated in neurological, infectious, autoimmune, and metabolic diseases, due to their high target specificity and cell-selective delivery, allowing targeted modulation of disease pathways while minimizing systemic toxicity. New advancements such as bispecific ADCs, dual-payload conjugates, and antibody-based delivery systems are utilized for nontraditional cargos. These advancements have greater potential to improve selectivity and overcome tumor heterogeneity than previous generations in the ADC format.

Despite these advances, the clinical translation of ADCs remains challenging, with several knowledge gaps limiting their broader application in patient care. A standardized PK/PD framework is critical to compare different ADCs, across various payload classes, linker types, and target categories in a reproducible manner. Another important concern is the inconsistencies reporting of adverse effects for class-specific toxicities (such as interstitial lung disease, ocular toxicity, and myelosuppression), which makes cross-study comparison difficult. Furthermore, there is insufficient data to support the biomarker strategies for patient selection, response prediction, and early toxicity detection, limiting its potential use as precision medicine. Overcoming these hurdles will require close collaboration among chemists, structural biologists, computational scientists, pharmacologists, and clinicians to align molecular design with manufacturability, translational feasibility, and clinical utility.

## Figures and Tables

**Table 1:** Summary of ADCs approved and in clinical trial.

ADC Type	ADC	Approved Year/Trial Phase	Target	Antibody Isotype	Linker Type	Cytotoxic Payload and Mechanism	DAR	Key Indications	NCT Number	References
**Hematologic**	Belantamab Mafodotin	Reapproved 2025	BCMA	IgG1	Non-cleavable	Monomethyl auristatin F (MMAF); tubulin polymerization inhibitor	4	R/R MM		[26,27,28,29]
	Zilovertamab Vedotin	Phase III	ROR1	IgG1	Cleavable	Monomethyl auristatin E (MMAE); tubulin polymerization inhibitor	4	DLBCL, R/R DLBCL	NCT06717347, NCT05139017	
**Solid Tumor**	Telisotuzumab Vedotin	2025	C-Met	IgG1	Cleavable	Monomethyl auristatin E (MMAE); tubulin polymerization inhibitor	3.1	la/mNSCLC with high C-Met		[30,31,32]
	ABBV-400/Telisotuzumab Adizutecan	Phase III	C-Met	IgG1	Cleavable	Adizutecan; topoisomerase I inhibitor	6	mCRC, la/mNSCLC,	NCT06614192, NCT07155187, NCT07005102	[33]
	Datopotamab Deruxtecan	2025	TROP2	IgG1	Cleavable	Deruxtecan (DXd); topoisomerase I inhibitor	4	HR+ HER- mBC, EGFR-mutated NSCLC		[34,35,36]
	FDA018	Phase III	TROP2	IgG1	Cleavable	SN-38; topoisomerase I inhibitor	7.6	la/r/mTNBC	NCT06519370	[3,37]
	MK-2870/Sacituzumab Tirumotecan	Phase III	TROP2	IgG1	Cleavable	KL610023; Topoisomerase I inhibitor	7.4	BC, TNBC, a/mNSCLC, UC, EC, OC, ps/rFTC, ps/rPPC, ps/rOC, HR+HER2- la/mBC, r/mCC, a/mGEC,	NCT06393374, NCT06074588, NCT07419295, NCT06132958, NCT07318558, NCT06170788, NCT06952504, NCT06841354, NCT06824467, NCT06312176, NCT06422143, NCT06459180, NCT07216703, NCT06312137, NCT06356311, NCT06966700, NCT06305754	[3,38,39]
	ARX-788/Anvatabart Opadotin	Phase II/III	HER2	IgG1	Non-cleavable	Monomethyl auristatin F (MMAF); tubulin polymerization inhibitor	1.9	HER2+BC	NCT05426486	[3]
	MRG002/Trastuzumab Vedotin	Phase III	HER2	IgG1	Cleavable	Monomethyl auristatin E (MMAE); tubulin polymerization inhibitor	3.8	la/mBC, a/mUC	NCT04924699, NCT05754853	[3]
	SYD985/Trastuzumab Duocarmazine	Phase III	HER2	IgG1	Cleavable	Seco-duobamycin hydroxybenzamide-azaindole (seco-DUBA); DNA alkylating agent	2.8	la/mBC	NCT03262935	[3,40]
	SHR-A1811/Trastuzumab Rezetecan	Phase III	HER2	IgG1	Cleavable	SHR9265; topoisomerase I inhibitor	5.7	HR+/HER2-BC, HER2+la/r/mBC, TNBC, rEOV, OV, aCRC, HER2+GC/GEJA, la/mGC, la/mGEJA, HER-NSCLC,	NCT07382687, NCT05424835, NCT06057610, NCT06990503, NCT06199973, NCT05814354, NCT06123494, NCT06828354, NCT07111832, NCT07196774, NCT06430437, NCT07118527, NCT06126640,	[3]
	BNT323/DB-1303	Phase III	HER2	IgG1	Cleavable	P1003; topoisomerase I inhibitor	8	HER2+BC, mBC, EC	NCT06265428, NCT06018337, NCT06340568	[3]
	DP303c/Trastuzumab Envedotin	Phase III	HER2	IgG1	Cleavable	Monomethyl auristatin E (MMAE); tubulin polymerization inhibitor	2	HER2+aBC	NCT06313086, NCT05901935	[3]
	FS-1502/Caxmotabart Entudotin	Phase III	HER2	IgG1	Cleavable	Monomethyl auristatin F (MMAF); tubulin polymerization inhibitor	2	la/mBC	NCT05755048	[41,42]
	BL-M07D1	Phase III	HER2	IgG1	Cleavable	Ed-04; topoisomerase I inhibitor	8	HER2+BC, HER+ la/mBC, HER2-low r/mBC, NSCLC, GC/GEJA	NCT06891833, NCT06830889, NCT06316531, NCT07152405, NCT07178795, NCT06957886	[43,44]
	A166/Trastuzumab Botidotin	Phase III	HER2	IgG1	Cleavable	Duo-5; tubulin polymerization inhibitor	2	HER+mBC	NCT06968585	[41,42]
	IBI354	Phase III	HER2	IgG1	-	Topoisomerase I inhibitor	8	prOC, prFTC, prPPC, HER+ mBC	NCT07377643, NCT06834672	[45]
	Patritumab Deruxtecan	Phase III	HER3	IgG1	Cleavable	Deruxtecan (DXd); topoisomerase I inhibitor	8	la/mNSCLC, BC	NCT05338970, NCT07060807	[3]
	ABT-414/Depatuxizumab Mafodotin	Phase III	EGFR	IgG1	Non-cleavable	Monomethyl auristatin F (MMAF); tubulin polymerization inhibitor	3.8	GBM, GSM	NCT02573324	[3]
	MRG003/Becotatug Vedotin	Phase III	EGFR	IgG1	Cleavable	Monomethyl auristatin E (MMAE); tubulin polymerization inhibitor	3.8	SCCHN, r/mNPC	NCT05751512, NCT06976190 NCT07459296	[3]
	SYS-6010	Phase III	EGFR	IgG1	Cleavable	JS-1; topoisomerase I inhibitor	8	NSCLC, la/mNSCLC, ESCC, la/r/mESCC, r/mBC	NCT07442565, NCT07376382, NCT07251062, NCT06927986, NCT07417735	[44,46]
	Raludotatug Deruxtecan	Phase II/III	CDH6	IgG1	Cleavable	Deruxtecan (DXd); topoisomerase I inhibitor	8	prOC, prFTC, prPPC	NCT06161025	[3]
	Ifinatamab Deruxtecan	Phase III	CD276	IgG1	Cleavable	Deruxtecan (DXd); topoisomerase I inhibitor	4	a/mESCC, SCLC, mPC	NCT06925737, NCT06203210, NCT06644781	[3,47]
	DB-1311/BNT324	Phase III	CD276	IgG1	Cleavable	P1021; Topoisomerase I inhibitor	6	mPC	NCT07365995	[48,49]
	MHB088C	Phase III	CD276	IgG1	Cleavable	Topoisomerase I Inhibitor	4	SCLC	NCT06954246	[44,49]
	HS-20093/GSK5764227	Phase III	CD276	IgG1	Cleavable	HS-9265; topoisomerase I inhibitor	4	SCLC, a/mNSCLC, OS	NCT06526624, NCT06498479, NCT07464327, NCT06935409, NCT07099898	[49,50]
	AZD8205/Puxitatug Samrotecan	Phase III	B7-H4	IgG1	Cleavable	AZ14170133; topoisomerase I inhibitor	8	EC	NCT07044336	[49,51]
	HS-20089/GSK5733584	Phase III	B7-H4	IgG1	Cleavable	HS-9265; topoisomerase I inhibitor	6	pr/rEOC, rEC, prOC	NCT06855069, NCT07286331, NCT07286266	[49,51,52]
	Rinatabart Sesutecan	Phase III	FRα	IgG1	Cleavable	Exatecan; topoisomerase I inhibitor	8	EC, rOC, prOC	NCT07166094, NCT07225270,NCT06619236	[3]
	AZD5335	Phase III	FRα	IgG1	Cleavable	AZ14170132; topoisomerase I inhibitor	8	prOC, prFTC, prPPC	NCT07218809	[53]
	LY4170156/Sofetabart Mipitecan	Phase III	FRα	IgG1	Cleavable	Exatecan; topoisomerase I inhibitor	8	prOC, psOC	NCT07213804	[54]
	ZL-1310/Zocilurtatug Pelitecan	Phase III	DLL3	IgG1	Cleavable	Topoisomerase I inhibitor	8	SCLC	NCT07218146	[55,56]
	SGN-PDL1V/PF-08046054	Phase III	PD-L1	IgG1	Cleavable	Monomethyl auristatin E (MMAE); tubulin polymerization inhibitor	-	NSCLC	NCT07144280	[57]
	SGN-B6/Sigvotatug Vedotin	Phase III	ITGB6	IgG1	Cleavable	Monomethyl auristatin E (MMAE); tubulin polymerization inhibitor	4	NSCLC	NCT06758401, NCT06012435	[3]
	AZD0901/Sonesitatug Vedotin	Phase III	CLDN18.2	IgG1	Cleavable	Monomethyl auristatin E (MMAE); tubulin polymerization inhibitor	4	a/mGC, a/mGEJA, a/mEAC	NCT06346392, NCT07431281	[58]
**Bispecific**	BL-B01D1	Phase III	EGFRxHER3	IgG1	Cleavable	Ed-04; topoisomerase I inhibitor	8	SCLC, NSCLC, EGFR-mutated NSCLC, UC, EOC, NPC, HR+HER2- BC, TNBC	NCT06500026, NCT06382129, NCT06838273, NCT06857175, NCT06382116, NCT06994195, NCT06304974, NCT06118333, NCT06382142, NCT06343948, NCT07100080, NCT07106762, NCT06926868	[59,60,61,62]
	JSKN003	Phase III	HER2xHER2	IgG1	Cleavable	Deruxtecan (DXd); Topoisomerase I inhibitor	4	prEOC, HER2+ aBC, HER2+ aCRC, HER2-low BC	NCT06751485, NCT06846437, NCT07384377, NCT06079983	[63,64,65,66]
	TQB 2102	Phase III	HER2xHER2	IgG1	Cleavable	Topoisomerase I Inhibitor	6	HER2+ aBC, HER2+ and HER2-low r/mBC	NCT07008976, NCT07003074, NCT07043725, NCT06561607	[3,67,68]

R/R: relapsed or refractory; la: locally advanced; r: recurrent; m: metastatic; pr: platinum resistant; ps: platinum sensitive; a: advanced; HR: hormone receptor; BC: breast cancer; TNBC: triple-negative breast cancer; MM: multiple myeloma; OC: ovarian cancer; EOC: epithelial ovarian cancer; EC: endometrial cancer; FTC: fallopian tube cancer; PPC: primary peritoneal cancer; CC: cervical cancer; SCLC: small cell lung cancer; PC: prostate cancer; ESCC: esophageal squamous cell carcinoma; CRC: colorectal cancer; GC: gastric cancer; GEC: gastroesophageal cancer; GEJA: gastroesophageal junction adenocarcinoma; EAC: esophageal adenocarcinoma; NSCLC: non-small cell lung cancer; GBM: glioblastoma; GSM: gliosarcoma; DLBCL: diffuse large B-cell lymphoma; SCCHN: squamous cell carcinoma of the head and neck; NPC: nasopharyngeal carcinoma; UC: urothelial cancer; OS: osteosarcoma.

**Table 2:** This mapping yields explicit engineering rules to guide next-generation development.

ADC Component/Design Choice	Associated Clinical Adverse Event (AE)	Underlying Mechanical Driver/Design Gap	Proposed Design Rule for Mitigation
High DAR (≥8) + Cleavable Linker + Lipophilic Payload (e.g., T-DXd)	Interstitial Lung Disease (ILD)/Pneumonitis	High systemic exposure caused by “bystander” payload leakage into highly vascularized alveolar spaces.	Optimize linker stability kinetics or utilize hydrophilic payloads to limit non-specific pulmonary tissue penetration.
High DAR + Microtubule Inhibitors (e.g., MMAE/Vedotin constructs)	Peripheral Neuropathy, Neutropenia	Premature systemic deconjugation/detachment of the payload disrupting microtubules in peripheral nerves and bone marrow.	Employ highly stable, site-specific conjugation technologies (e.g., THIOMAB) to minimize off-target free payload circulation.
Maytansinoid Payloads (e.g., DM4)	Ocular Toxicities (Keratopathy/Blurred Vision)	Non-specific uptake of the circulating ADC or free payload into corneal epithelial cells via Macropinocytosis.	Engineer conditionally activated “masked” antibodies (Pro-ADCs) that selectively unmask within the protease-rich tumor microenvironment.

## Data Availability

No new data was created or analyzed in this study.

## References

[1] Wu G., Yuan Z., Chen M., Tang X., Wang F., Zhang D. (2026). Antibody–Drug Conjugates (ADCs): A Review of Structural Design, Technological Evolution, and Future Perspectives. Molecules.

[2] Cheng B., Gong L., Wang Z., Peng P., Xu K., Huang H., Wu P. (2026). Antibody–Drug Conjugates in Oncology: Principles, Clinical Development, and Future Directions. MedComm.

[3] Wang R., Hu B., Pan Z., Mo C., Zhao X., Liu G., Hou P., Cui Q., Xu Z., Wang W. (2025). Antibody-Drug Conjugates (ADCs): Current and future biopharmaceuticals. J. Hematol. Oncol..

[4] Kurtova A.I., Iureva A.M., Esetov N.S., Sokol D.V., Fedotova P.A., Shipunova V.O. (2026). Challenges and opportunities beyond antibody-drug conjugates: Toward a new era of programmable therapeutic proteins. Biochem. Biophys. Res. Commun..

[5] Buonaguro L., Tagliamonte M. (2020). Selecting Target Antigens for Cancer Vaccine Development. Vaccines.

[6] Abdeldaim D.T., Schindowski K. (2023). Fc-Engineered Therapeutic Antibodies: Recent Advances and Future Directions. Pharmaceutics.

[7] Li S., Guo Y., Che J., Dai H., Dong X. (2025). Recent Advances in Peptide Linkers for Antibody-Drug Conjugates. J. Med. Chem..

[8] Izzo D., Ascione L., Guidi L., Marsicano R.M., Koukoutzeli C., Trapani D., Curigliano G. (2025). Innovative payloads for ADCs in cancer treatment: Moving beyond the selective delivery of chemotherapy. Ther. Adv. Med. Oncol..

[9] Fan Q., Chen H., Wei G., Wei D., Wang Z., Zhang L., Wang J., Zhu M. (2025). A review of conjugation technologies for antibody drug conjugates. Antib. Ther..

[10] Manabe S., Senga S., Iwamoto S., Yarita H., Takahashi R., Mitani A., Sumiyoshi W., Hoshinoo A., Hiranyakorn M., Kinoshita T. (2026). Site-Specific Glycan Conjugation Improves Stability and Efficacy of an Antibody–Drug Conjugates Bearing DXd as a Cytotoxic Payload. ACS Omega.

[11] Aggarwal D., Yang J., Salam M.A., Sengupta S., Al-Amin M.Y., Mustafa S., Khan M.A., Huang X., Pawar J.S. (2023). Antibody-drug conjugates: The paradigm shifts in the targeted cancer therapy. Front. Immunol..

[12] Chau C.H., Steeg P.S., Figg W.D. (2019). Antibody–drug conjugates for cancer. Lancet.

[13] Esapa B., Jiang J., Cheung A., Chenoweth A., Thurston D.E., Karagiannis S.N. (2023). Target Antigen Attributes and Their Contributions to Clinically Approved Antibody-Drug Conjugates (ADCs) in Haematopoietic and Solid Cancers. Cancers.

[14] Tsao L.-C., Wang J.S., Ma X., Sodhi S., Ragusa J.V., Liu B., McBane J., Wang T., Wei J., Liu C.-X. (2025). Effective extracellular payload release and immunomodulatory interactions govern the therapeutic effect of trastuzumab deruxtecan (T-DXd). Nat. Commun..

[15] Modi S., Jacot W., Yamashita T., Sohn J., Vidal M., Tokunaga E., Tsurutani J., Ueno N.T., Prat A., Chae Y.S. (2022). Trastuzumab Deruxtecan in Previously Treated HER2-Low Advanced Breast Cancer. New Engl. J. Med..

[16] Yi M., Li T., Gu Y., Niu M., Xue D., Hu S., Wu Y., Zhao B., Zhang D., Ma Y. (2026). Triple targeting of STING, TGF-β, and PD-L1 boosts CXCL16–CXCR6 signaling for potent antitumor response. Nat. Commun..

[17] Yi M., Zhang J., Li A., Niu M., Yan Y., Jiao Y., Luo S., Zhou P., Wu K. (2021). The construction; expression, and enhanced anti-tumor activity of YM101: A bispecific antibody simultaneously targeting TGF-β and PD-L1. J. Hematol. Oncol..

[18] Hoffmann R.M., Coumbe B.G.T., Josephs D.H., Mele S., Ilieva K.M., Cheung A., Tutt A.N., Spicer J.F., Thurston D.E., Crescioli S. (2018). Antibody structure and engineering considerations for the design and function of Antibody Drug Conjugates (ADCs). Oncoimmunology.

[19] (1993). Bylaws. American Association for Medical Transcription. J. Am. Assoc. Med. Transcr..

[20] Zhang Y., Sun L., Ren S., Pan F., Zhu H., Dang Q., Tian P., Liu F., Wu L., Guo S. (2025). Phase 1 study of SHR-1826, a c-MET–directed antibody-drug-conjugate (ADC), in advanced solid tumors. J. Clin. Oncol..

[21] Liu K., Li M., Li Y., Li Y., Chen Z., Tang Y., Yang M., Deng G., Liu H. (2024). A review of the clinical efficacy of FDA-approved antibody-drug conjugates in human cancers. Mol. Cancer.

[22] Hungria V., Robak P., Hus M., Zherebtsova V., Ward C., Ho P.J., de Almeida A.C.R., Hajek R., Kim K., Grosicki S. (2024). Mateos, Belantamab Mafodotin, Bortezomib, and Dexamethasone for Multiple Myeloma. N. Engl. J. Med..

[23] Sun Y., Yu X., Wang X., Yuan K., Wang G., Hu L., Zhang G., Pei W., Wang L., Sun C. (2023). Bispecific antibodies in cancer therapy: Target selection and regulatory requirements. Acta Pharm. Sin. B.

[24] Sung H., Siegel R.L., Laversanne M., Jiang C., Morgan E., Zahwe M., Cao Y., Bray F., Jemal A. (2025). Colorectal cancer incidence trends in younger versus older adults: An analysis of population-based cancer registry data. Lancet Oncol..

[25] Zhang Y., Du J., Cui X., Ling Y., Tang C. (2025). Development of a bispecific CDH17-GUCY2C ADC bearing the ferroptosis inducer RSL3 for the treatment of colorectal cancer. Cell Death Discov..

[26] Fu Z., Li S., Han S., Shi C., Zhang Y. (2022). Antibody drug conjugate: The “biological missile” for targeted cancer therapy. Signal Transduct. Target. Ther..

[27] Ketchum E.B., Clarke A., Clemmons A.B. (2022). Belantamab Mafodotin-blmf: A Novel Antibody-Drug Conjugate for Treatment of Patients with Relapsed/Refractory Multiple Myeloma. J. Adv. Pract. Oncol..

[28] Trudel S., Lendvai N., Popat R., Voorhees P.M., Reeves B., Libby E.N., Richardson P.G., Anderson L.D., Sutherland H.J., Yong K. (2018). Targeting B-cell maturation antigen with GSK2857916 antibody-drug conjugate in relapsed or refractory multiple myeloma (BMA117159): A dose escalation and expansion phase 1 trial. Lancet Oncol..

[29] Hungria V., Robak P., Hus M., Zherebtsova V., Ward C., Ho P.J., Hájek R., Kim K., Grosicki S., Sia H. (2025). Belantamab mafodotin plus bortezomib and dexamethasone in patients with relapsed or refractory multiple myeloma (DREAMM-7): Updated overall survival analysis from a global, randomised, open-label, phase 3 trial. Lancet Oncol..

[30] Camidge D.R., Bar J., Horinouchi H., Goldman J., Moiseenko F., Filippova E., Cicin I., Ciuleanu T., Daaboul N., Liu C. (2024). Telisotuzumab Vedotin Monotherapy in Patients with Previously Treated c-Met Protein-Overexpressing Advanced Nonsquamous EGFR-Wildtype Non-Small Cell Lung Cancer in the Phase II LUMINOSITY Trial. J. Clin. Oncol..

[31] Blair H.A. (2025). Telisotuzumab Vedotin: First Approval. Drugs.

[32] Zhao C., Lu D., Gao J. (2025). Telisotuzumab vedotin: The first-in-class c-Met-targeted antibody-drug conjugate granted FDA accelerated approval for treatment of non-squamous non-small cell lung cancer (NSCLC). Drug Discov. Ther..

[33] Wang Y., Lu K., Xu Y., Xu S., Chu H., Fang X. (2025). Antibody-drug conjugates as immuno-oncology agents in colorectal cancer: Targets, payloads, and therapeutic synergies. Front. Immunol..

[34] Blair H.A. (2025). Datopotamab Deruxtecan: First Approval. Drugs.

[35] Okajima D., Yasuda S., Maejima T., Karibe T., Sakurai K., Aida T., Toki T., Yamaguchi J., Kitamura M., Kamei R. (2021). Datopotamab Deruxtecan, a Novel TROP2-directed Antibody-drug Conjugate, Demonstrates Potent Antitumor Activity by Efficient Drug Delivery to Tumor Cells. Mol. Cancer Ther..

[36] Royce M., Shah M., Zhang L., Cheng J., Bonner M.K., Pegues M., Miller C.P., Leu L., Price L.S.L., Qiu J. (2025). FDA Approval Summary: Datopotamab Deruxtecan-dlnk for Treatment of Patients with Unresectable or Metastatic, HR-Positive, HER2-Negative Breast Cancer. Clin. Cancer Res..

[37] Cheng M., Li X., Li Y., Wang Y., Li W., Wang S., Shu C., Song Q., Ding L. (2025). LBA and LC-MS/MS based comprehensive bioanalytical methods for FDA018, a Trop-2 targeted antibody-drug conjugate. J. Pharm. Biomed. Anal..

[38] Sofianidi A.A., Papatheodoridi A., Dimitrakakis C., Marinopoulos S., Michalaki V., Zagouri F. (2026). Sacituzumab tirumotecan (sac-TMT/MK-2870/SKB264): A novel antibody–drug conjugate in breast cancer. Oncol. Rev..

[39] Zhang L., Fang W., Cheng Y., Meng X., Yang R., Jiang G., Cui J., He L., Chen P., Zheng W. (2025). Sacituzumab tirumotecan (sac-TMT) in patients (pts) with previously treated locally advanced or metastatic (LA/M) non-small cell lung cancer (NSCLC) harboring uncommon EGFR mutations: Preliminary results from a phase 2 study. J. Clin. Oncol..

[40] Menderes G., Bonazzoli E., Bellone S., Black J., Predolini F., Pettinella F., Masserdotti A., Zammataro L., Altwerger G., Buza N. (2017). SYD985, a Novel Duocarmycin-Based HER2-Targeting Antibody-Drug Conjugate, Shows Antitumor Activity in Uterine and Ovarian Carcinosarcoma with HER2/Neu Expression. Clin. Cancer Res..

[41] Qiu Y., Shi Y., Chao Z., Zhu X., Chen Y., Lu L. (2025). Recent advances of antibody-drug conjugates in treating breast cancer with different HER2 status. Ther. Adv. Med. Oncol..

[42] Zhang X., Huang A.C., Chen F., Chen H., Li L., Kong N., Luo W., Fang J. (2022). Novel development strategies and challenges for anti-Her2 antibody-drug conjugates. Antib. Ther..

[43] Zimmerman B.S., Esteva F.J. (2024). Next-Generation HER2-Targeted Antibody-Drug Conjugates in Breast Cancer. Cancers.

[44] Wu D., Yang K., He R., Yin R., Shui L. (2025). Antibody-drug conjugates in cancer therapy: Current advances and prospects for breakthroughs. Front. Cell Dev. Biol..

[45] Shu J., Zhu T., Huang Y., Xu Q., Guo R., Liu H., Zhao H., Zhu L., Wang X., Xu X. (2025). IBI354, an anti-HER2 antibody-drug conjugate, in patients with locally advanced unresectable or metastatic ovarian cancers: Updated results from a phase I trial. J. Clin. Oncol..

[46] Lu S., Zhou Z., Li Z.-M., He Z.-Y., Fang J., Sun H.-M., Gong Y., Cheng Y., Liu Y.-B., Yao Y. (2025). Abstract CT008: First-in-human study of SYS6010, a novel EGFR targeting antibody drug conjugate (ADC) for patients with advanced solid tumors. Cancer Res..

[47] Yamato M., Hasegawa J., Maejima T., Hattori C., Kumagai K., Watanabe A., Nishiya Y., Shibutani T., Aida T., Hayakawa I. (2022). DS-7300a, a DNA Topoisomerase I Inhibitor, DXd-Based Antibody-Drug Conjugate Targeting B7-H3, Exerts Potent Antitumor Activities in Preclinical Models. Mol. Cancer Ther..

[48] Schoenfeld A., Spira A., Lisberg A.E., Gutierrez M., Alexander M., Sankhala K., Cheng Y., Escriu C., Forster M., Kao S. (2025). P3.18.74 BNT324-01: A Phase 1b/2 Trial of BNT324/DB-1311 (B7H3 ADC) with BNT327 (PD-L1 x VEGF-A bsAb) in Lung Cancer (SCLC or NSCLC). J. Thorac. Oncol..

[49] Brown E.F., Colombo I., Madariaga A., Kasherman L. (2025). Immunological mechanisms and antibody-drug conjugates targeting B7-H3 and B7-H4 in ovarian cancer. Front. Immunol..

[50] Duan J., Sun Y., Wang Q., Xing L., Wu L., Han L., Chen J., Liu B., Lu P., Shi H. (2026). HS-20093, a B7-H3-targeted antibody-drug conjugate in lung cancer: Results from the ARTEMIS-001 phase 1a/b trial. Cancer Cell.

[51] Marchesi S., Marinello A., Ambrosini P., Cavalli C., Russo G.L., Occhipinti M. (2025). Immune-checkpoint targeting drug conjugates: A novel class of promising therapeutic agents for cancer treatment. npj Precis. Oncol..

[52] Tu P., Li G., Zou W., Xu C., Wang J. (2025). Advances in Antibody–Drug Conjugates for Endometrial Cancer. Mol. Cancer Ther..

[53] Zoeller J.J., Tammali R., Dodd R.B., Patel N.V., Andoni A., Bisha I., Cronin S., De Almeida A., Lee N., Meekin J.H. (2025). Derivation of AZD5335, a Novel FRα-Targeted TOP1i-Loaded ADC, for the Treatment of FRα-Expressing Cancers. Clin. Cancer Res..

[54] Ray-Coquard I.L., O’Malley D.M., Pothuri B., Oaknin A., Lakhani N.J., Koyama T., Kitano S., McKean W.B., Madariaga A., Okera M. (2025). Initial results from a first-in-human phase 1 study of LY4170156, an ADC targeting folate receptor alpha (FRα), in advanced ovarian cancer and other solid tumors. J. Clin. Oncol..

[55] Zlatanova T., Changalova A., Janzic U. (2026). The role of antibody-drug conjugates in the treatment of lung cancer. Front. Oncol..

[56] Lin L.N., Wan B., Ye Q., Wang L., Wang C., Peng W., Wang J., Dai X., Chen M., Lv C. (2024). 241P Development and characterization of a novel DLL3-targeting antibody drug conjugate (ADC) for the treatment of solid tumors. ESMO Open.

[57] Kwan B., Ramirez M., Jin S., Yu C., Wo S., Gupta P., Allred S., Simmons J., Hensley K., de Zafra C.Z. (2021). 783 SGN-PDL1V, a novel, investigational PD-L1-directed antibody-drug conjugate for the treatment of solid tumors, in: Regular and Young Investigator Award Abstracts. J. Immunother. Cancer.

[58] Ruan D.-Y., Liu F.-R., Wei X.-L., Luo S.-X., Zhuang Z.-X., Wang Z.-N., Liu F.-N., Zhang Y.-Q., Yang J.-W., Chen Z.-D. (2025). Claudin 18.2-targeting antibody-drug conjugate CMG901 in patients with advanced gastric or gastro-oesophageal junction cancer (KYM901): A multicentre, open-label, single-arm, phase 1 trial. Lancet Oncol..

[59] Wan W., Zhao S., Zhuo S., Zhang Y., Chen L., Li G., Renshaw B., Khalili J.S., Xiao S., Zhu Y. (2023). Abstract 2642: BL-B01D1, a novel EGFR×HER3-targeting ADC, demonstrates robust anti-tumor efficacy in preclinical evaluation. Cancer Res..

[60] Liu C., Liu D., Ji Y., Sun M., Gao S., Ma X., Zhong D., Zhu J., Cao Y., Qi C. (2025). A bispecific antibody-drug conjugate targeting EGFR and HER3 in metastatic esophageal squamous cell carcinoma: A phase 1b trial. Nat. Med..

[61] Gu Y., Wang Z., Wang Y. (2024). Bispecific antibody drug conjugates: Making 1 + 1 > 2. Acta Pharm. Sin. B.

[62] Lu Z., Chang L., Zhou J., Ji Y., Sun M., Wen Q., Gao S.G., Ma X.L., Zhong D., Guo Q. (2024). 54P BL-B01D1, an EGFR x HER3 bispecific antibody-drug conjugate (ADC), in patients with locally advanced or metastatic biliary tract carcinoma (BTC). Ann. Oncol..

[63] Wang P., Guo K., Peng J., Sun J., Xu T. (2023). JSKN003, a Novel biparatopic anti-Her2 antibody-drug conjugate, exhibits potent antitumor efficacy. Antib. Ther..

[64] Zhang Q., Wang J., Ouyang Q., Wang X., Lin D., Wang J., Gan L., Ouyang Z., Xu T., Liu Y. (2023). 418P Two-year follow-up data on the efficacy and safety of KN026, a HER2-targeted bispecific antibody combined with docetaxel as first-line treatment for HER2-positive recurrent/metastatic breast cancer. Ann. Oncol..

[65] Wu X., Chen Y., Rao Q., Li J., Gao B., Weng G., Zhang Z., Lan C., Tang D., Wilkinson K.J. (2025). JSKN003, a biparatopic anti-HER2 antibody drug conjugate (ADC), in the treatment of platinum-resistant ovarian cancer (PROC): Updated findings from two clinical trials. J. Clin. Oncol..

[66] Liu D., Liu B., Zhang X., Ruan J., Weng G., Zhang Z., Li Q., Zong H., Dai J., Xu T. (2025). 806P Efficacy and safety of JSKN003: A biparatopic anti-HER2 antibody conjugate (ADC), in patients with HER2-positive metastatic colorectal cancer (mCRC). Ann. Oncol..

[67] Zhang L., Wang Q., Yang Y., Huang D., Yao Y., Zhang J., Sun L., Guo R., Ma S., Wang L. (2025). PT2.10.04 Efficacy and Safety of TQB2102, a Biparatopic HER2-Targeting ADC, in HER-2 Aberrant NSCLC: A Phase II Study. J. Thorac. Oncol..

[68] Li J.-J., Zhang W.-J., Zeng X.-H., Zhang Q.-Y., Chen L., Wu J., Liu G.-Y., Wang Z.-H., Hu X.-B., Hu Y.-Y. (2026). Efficacy and Safety of Neoadjuvant TQB2102 in Locally Advanced or Early Human Epidermal Growth Factor Receptor 2-Positive Breast Cancer: A Randomized, Open-Label, Multicenter, Phase II Trial. J. Clin. Oncol..

[69] Blank L., Pander G., Mühlberg E., Mier W., Uhl P. (2025). Exploiting the unique properties of nanobodies: Enhancing therapeutics, drug delivery, and targeted diagnostics. Drug Discov. Today.

[70] van Faassen H., Ryan S., Henry K.A., Raphael S., Yang Q., Rossotti M.A., Brunette E., Jiang S., Haqqani A.S., Sulea T. (2020). Serum albumin-binding V _H_ Hs with variable pH sensitivities enable tailored half-life extension of biologics. FASEB J..

[71] Ishiwatari-Ogata C., Kyuuma M., Ogata H., Yamakawa M., Iwata K., Ochi M., Hori M., Miyata N., Fujii Y. (2022). Ozoralizumab, a Humanized Anti-TNFα NANOBODY® Compound, Exhibits Efficacy Not Only at the Onset of Arthritis in a Human TNF Transgenic Mouse but Also During Secondary Failure of Administration of an Anti-TNFα IgG. Front. Immunol..

[72] Teunissen A.J., Abousaway O.B., Munitz J., van Leent M.M., Toner Y.C., Priem B., Senders M.L., Pérez-Medina C., Mulder W.J., Rashidian M. (2021). Employing nanobodies for immune landscape profiling by PET imaging in mice. STAR Protoc..

[73] Wu J., Lu H., Xu X., Rao L., Ge Y. (2024). Engineered Cellular Vesicles Displaying Glycosylated Nanobodies for Cancer Immunotherapy. Angew. Chem. Int. Ed. Engl..

[74] Van Butsel B., Sargentini-Maier M.L., Marques A.P., Vandenbossche Y., Marcheva G., Gunawardena S., Pine S. (2024). Complex immunogenicity assessment in caplacizumab-treated patients with immune-mediated thrombotic thrombocytopenic purpura who have received plasma exchange. Res. Pract. Thromb. Haemost..

[75] Takeuchi T., Chino Y., Kawanishi M., Nakanishi M., Watase H., Mano Y., Sato Y., Uchida S., Tanaka Y. (2023). Efficacy and pharmacokinetics of ozoralizumab, an anti-TNFα NANOBODY® compound, in patients with rheumatoid arthritis: 52-week results from the OHZORA and NATSUZORA trials. Arthritis Res. Ther..

[76] Gerber H.-P., Gangwar S., Betts A. (2023). Therapeutic index improvement of antibody-drug conjugates. MAbs.

[77] Conilh L., Sadilkova L., Viricel W., Dumontet C. (2023). Payload diversification: A key step in the development of antibody–drug conjugates. J. Hematol. Oncol..

[78] Sheyi R., de la Torre B.G., Albericio F. (2022). Linkers: An Assurance for Controlled Delivery of Antibody-Drug Conjugate. Pharmaceutics.

[79] Balamkundu S., Liu C.-F. (2023). Lysosomal-Cleavable Peptide Linkers in Antibody–Drug Conjugates. Biomedicines.

[80] de Almeida V.M., Soares M.B.P., Santos-Filho O.A. (2025). Exploring Experimental and In Silico Approaches for Antibody–Drug Conjugates in Oncology Therapies. Pharmaceuticals.

[81] Matikonda S.S., McLaughlin R., Shrestha P., Lipshultz C., Schnermann M.J. (2022). Structure–Activity Relationships of Antibody-Drug Conjugates: A Systematic Review of Chemistry on the Trastuzumab Scaffold. Bioconjug. Chem..

[82] Wang Z., Li H., Gou L., Li W., Wang Y. (2023). Antibody–drug conjugates: Recent advances in payloads. Acta Pharm. Sin. B.

[83] Han S., Lim K.S., Blackburn B.J., Yun J., Putnam C.W., Bull D.A., Won Y.-W. (2022). The Potential of Topoisomerase Inhibitor-Based Antibody–Drug Conjugates. Pharmaceutics.

[84] Khera E., Dong S., Huang H., de Bever L., van Delft F.L., Thurber G.M. (2022). Cellular-Resolution Imaging of Bystander Payload Tissue Penetration from Antibody-Drug Conjugates. Mol. Cancer Ther..

[85] Tolosa E.J., Yang L., Ayers-Ringler J., Suzuki S., Mallareddy J.R., Schaefer-Klein J., Borad M., Kosari F., Natarajan A., Mansfield A.S. (2024). Proteolysis targeting chimera (PROTAC)-driven antibody internalization of oncogenic cell surface receptors. Commun. Biol..

[86] Wen M., Yu A., Park Y., Calarese D., Gerber H.-P., Yin G. (2025). Homogeneous antibody-drug conjugates with dual payloads: Potential, methods and considerations. MAbs.

[87] Ren P., Guan M., Tang J., Yin S., Qi L., Yue J., Li Z., Fan X., Lei G., Zuo T. (2026). A Novel Dual-Payload ADC Platform Integrating Exatecan and Triptolide to Enhance Antitumor Efficacy and Overcome Resistance. Mol. Cancer Ther..

[88] Beerli R.R., Hell T., Merkel A.S., Grawunder U. (2015). Sortase Enzyme-Mediated Generation of Site-Specifically Conjugated Antibody Drug Conjugates with High In Vitro and In Vivo Potency. PLoS ONE.

[89] Thoreau F., Rochet L.N.C., Baker J.R., Chudasama V. (2023). Enabling the formation of native mAb, Fab′ and Fc-conjugates using a bis-disulfide bridging reagent to achieve tunable payload-to-antibody ratios (PARs). Chem. Sci..

[90] Ochtrop P., Hackenberger C.P.R. (2020). Recent advances of thiol-selective bioconjugation reactions. Curr. Opin. Chem. Biol..

[91] Szijj P.A., Bahou C., Chudasama V. (2018). Minireview: Addressing the retro-Michael instability of maleimide bioconjugates. Drug Discov. Today Technol..

[92] Dudchak R., Podolak M., Holota S., Szewczyk-Roszczenko O., Roszczenko P., Bielawska A., Lesyk R., Bielawski K. (2024). Click chemistry in the synthesis of antibody-drug conjugates. Bioorg. Chem..

[93] Kasper M., Stengl A., Ochtrop P., Gerlach M., Stoschek T., Schumacher D., Helma J., Penkert M., Krause E., Leonhardt H. (2019). Hackenberger, Ethynylphosphonamidates for the Rapid and Cysteine-Selective Generation of Efficacious Antibody–Drug Conjugates. Angew. Chem. Int. Ed..

[94] Wang Y., Xie F., Liu L., Xu X., Fan S., Zhong W., Zhou X. (2022). Development of applicable thiol-linked antibody–drug conjugates with improved stability and therapeutic index. Drug Deliv..

[95] Benjamin S.R., Jackson C.P., Fang S., Carlson D.P., Guo Z., Tumey L.N. (2019). Thiolation of Q295: Site-Specific Conjugation of Hydrophobic Payloads without the Need for Genetic Engineering. Mol. Pharm..

[96] Kishimoto S., Nakashimada Y., Yokota R., Hatanaka T., Adachi M., Ito Y. (2019). Site-Specific Chemical Conjugation of Antibodies by Using Affinity Peptide for the Development of Therapeutic Antibody Format. Bioconjug. Chem..

[97] Matsuda Y., Chang J.R., Mendelsohn B.A. (2025). Advanced Antibody–Drug Conjugates Design: Innovation in Linker Chemistry and Site-Specific Conjugation Technologies. ChemBioChem.

[98] Matsuda Y., Shikida N., Hatada N., Yamada K., Seki T., Nakahara Y., Endo Y., Shimbo K., Takahashi K., Nakayama A. (2024). AJICAP-M: Traceless Affinity Peptide Mediated Conjugation Technology for Site-Selective Antibody–Drug Conjugate Synthesis. Org. Lett..

[99] Fujii T., Matsuda Y., Seki T., Shikida N., Iwai Y., Ooba Y., Takahashi K., Isokawa M., Kawaguchi S., Hatada N. (2023). AJICAP Second Generation: Improved Chemical Site-Specific Conjugation Technology for Antibody–Drug Conjugate Production. Bioconjug. Chem..

[100] Adhikari P., Zacharias N., Ohri R., Sadowsky J. (2020). Site-Specific Conjugation to Cys-Engineered THIOMAB^TM^ Antibodies. Methods Mol. Biol..

[101] Liao X., Haight A., Welch D., Han L. (2023). Selective Reduction of Cysteine Mutant Antibodies for Site-Specific Antibody–Drug Conjugates. Bioconjugate Chem..

[102] Cortés J., Punie K., Barrios C., Hurvitz S.A., Schneeweiss A., Sohn J., Tokunaga E., Brufsky A., Park Y.H., Xu B. (2025). Sacituzumab Govitecan in Untreated, Advanced Triple-Negative Breast Cancer. New Engl. J. Med..

[103] Alradwan I.A., Alnefaie M.K., AL Fayez N., Aodah A.H., Majrashi M.A., Alturki M., Fallatah M.M., Almughem F.A., Tawfik E.A., Alshehri A.A. (2025). Strategic and Chemical Advances in Antibody–Drug Conjugates. Pharmaceutics.

[104] Shi X., Tang K., Zhang Q., Han Q., Quan L., Li Y., Cui J., Feng N., Gong J., Shang B. (2025). Antibody-drug conjugate combinations in cancer treatment: Clinical efficacy and clinical study perspectives. Front. Pharmacol..

[105] Tumey L.N., Li F., Rago B., Han X., Loganzo F., Musto S., Graziani E.I., Puthenveetil S., Casavant J., Marquette K. (2017). Site Selection: A Case Study in the Identification of Optimal Cysteine Engineered Antibody Drug Conjugates. AAPS J..

[106] Hingorani D.V. (2024). An overview of site-specific methods for achieving antibody drug conjugates with homogenous drug to antibody ratio. Expert Opin. Biol. Ther..

[107] Zhang W., Wang H., Feng N., Li Y., Gu J., Wang Z. (2023). Developability assessment at early-stage discovery to enable development of antibody-derived therapeutics. Antib. Ther..

[108] Hobson A.D., Xu J., Marvin C.C., McPherson M.J., Hollmann M., Gattner M., Dzeyk K., Fettis M.M., Bischoff A.K., Wang L. (2023). Optimization of Drug-Linker to Enable Long-term Storage of Antibody–Drug Conjugate for Subcutaneous Dosing. J. Med. Chem..

[109] Zhao R.Y., Wilhelm S.D., Audette C., Jones G., Leece B.A., Lazar A.C., Goldmacher V.S., Singh R., Kovtun Y., Widdison W.C. (2011). Synthesis and Evaluation of Hydrophilic Linkers for Antibody–Maytansinoid Conjugates. J. Med. Chem..

[110] Wen L., Zhang Y., Sun C., Wang S.S., Gong Y., Jia C., Luo J. (2025). Fundamental properties and principal areas of focus in antibody–drug conjugates formulation development. Antib. Ther..

[111] Abdou H.A., Awny S. (2026). Antibody-Drug Conjugates in Solid Tumors: Mechanisms, Clinical Advances, and Emerging Resistance Patterns. ASIDE Oncol..

[112] Pfeifer M., Zheng B., Erdmann T., Koeppen H., McCord R., Grau M., Staiger A., Chai A., Sandmann T., Madle H. (2015). Anti-CD22 and anti-CD79B antibody drug conjugates are active in different molecular diffuse large B-cell lymphoma subtypes. Leukemia.

[113] Xu J., Luo W., Li C., Mei H. (2023). Targeting CD22 for B-cell hematologic malignancies. Exp. Hematol. Oncol..

[114] Lisignoli G., Toneguzzi S., Piacentini A., Cattini L., Lenti A., Tschon M., Cristino S., Grassi F., Facchini A. (2003). Human osteoblasts express functional CXC chemokine receptors 3 and 5: Activation by their ligands, CXCL10 and CXCL13, significantly induces alkaline phosphatase and beta-N-acetylhexosaminidase release. J. Cell. Physiol..

[115] Dong Y., Zhi Y., Li X., Ma M., Ye M., Huang S., Tang J., Zhong W., Lei X., Mao Y. (2025). Navigating hepatotoxicity of antibody-drug conjugates: From mechanistic insights to clinical and postmarketing evidence. Front. Pharmacol..

[116] Hao Y., Song Z. (2025). Mechanisms of resistance to antibody-drug conjugates in cancer therapy: Molecular basis and therapeutic strategies. Cancer Drug Resist..

[117] Abelman R.O., Wu B., Spring L.M., Ellisen L.W., Bardia A. (2023). Mechanisms of Resistance to Antibody–Drug Conjugates. Cancers.

[118] Larose É.A., Hua X., Yu S., Pillai A.T., Yi Z., Yu H. (2025). Antibody-drug conjugates in breast cancer treatment: Resistance mechanisms and the role of therapeutic sequencing. Cancer Drug Resist..

[119] Takahashi N., Surolia I., Thomas A. (2020). Targeting DNA Repair to Drive Immune Responses: It’s Time to Reconsider the Strategy for Clinical Translation. Clin. Cancer Res..

[120] Azhar M.S.M., Loh Z., Mutamba F.T., Algahiny A., Yunusa N.M., Paramasevon S., Almusarhed M. (2025). Emerging Therapeutic Synergies: Combining PD-1 Inhibitors with Poly-ADP-Ribose Polymerase (PARP) Inhibitors in the Treatment of Gynecologic Cancers. Cureus.

[121] Lu Y., Huang W., Li Y., Xu Y., Wei Q., Sha C., Guo P. (2025). Leveraging artificial intelligence in antibody-drug conjugate development: From target identification to clinical translation in oncology. npj Precis. Oncol..

[122] Fang J., Guo L., Zhang Y., Guo Q., Wang M., Wang X. (2024). The target atlas for antibody-drug conjugates across solid cancers. Cancer Gene Ther..

[123] Yap M., Mihai I.-M., Wang G. (2026). Machine Learning in Biomarker-Driven Precision Oncology: Automated Immunohistochemistry Scoring and Emerging Directions in Genitourinary Cancers. Curr. Oncol..

[124] Noriega H.A., Wang X.S. (2025). AI-driven innovation in antibody-drug conjugate design. Front. Drug Discov..

[125] Wang Y., Guo C., Li W. (2025). Artificial intelligence in antibody–drug conjugate development. Trends Pharmacol. Sci..

[126] Zhou Q. (2017). Site-Specific Antibody Conjugation for ADC and Beyond. Biomedicines.

[127] Arsiwala A., Bhatt R., van Niekerk L., Quintero-Cadena P., Ao X., Rosenbaum A., Bhatt A., Smith A., Yang Y., Anderson K. (2025). A high-throughput platform for biophysical antibody developability assessment to enable AI/ML model training. MAbs.

[128] Wang Z., Zhu J., Sang L., Tang L., Zhang S., Tan Y., Zhao Y., Hao K. (2025). PBPK-PD model for predicting pharmacokinetics, tumor growth inhibition, and toxicity risks of topoisomerase inhibitor ADCs in mice and humans. Eur. J. Pharm. Sci..

[129] Li Y., Wilkins A.K., Davis J., Knab T., Toukam M., Boni J.P., Kirouac D.C. (2025). QSP modeling of loncastuximab tesirine with T-cell-dependent bispecific antibodies guides dose-regimen strategy. npj Syst. Biol. Appl..

[130] Zhang R., Wen H., Lin Z., Li B., Zhou X. (2025). Artificial Intelligence-Driven Drug Toxicity Prediction: Advances, Challenges, and Future Directions. Toxics.

[131] Eckardt J.-N., Wendt K., Bornhäuser M., Middeke J.M. (2021). Reinforcement Learning for Precision Oncology. Cancers.

[132] Wali A.F., El-Tanani M., Talath S., Rabbani S.A., Rangraze I.R., Satyam S.M., Avagimyan A., Hoffmann K., Ilias I., Ispas S. (2025). Antibody-Drug Conjugates and Beyond: Next-Generation Targeted Therapies for Breast Cancer. Cancers.

[133] Fu C. (2025). Where does ISAC (immune-stimulating antibody conjugates) go from here?. J. Immunother. Cancer.

